# Supplementary material for: DNA mechanotechnology reveals that integrin receptors apply pN forces in podosomes on fluid substrates
Source: Nat Commun. 2019 Oct 18;10:4507. doi: 10.1038/s41467-019-12304-4 (PMC6800454; doi:10.1038/s41467-019-12304-4)
Supplement: Supplementary file 1 — Supplementary Information [file 41467_2019_12304_MOESM1_ESM.pdf]

Supplementary Information

DNA mechanotechnology reveals that integrin receptors apply pN forces in podosomes on fluid substrates

Roxanne Glazier[1], Joshua M. Brockman[1], Emily Bartle[2], Alexa Mattheyses[2], Olivier Destaing*[3], Khalid Salaita*[1,4]

[1]*Wallace H. Coulter Department of Biomedical Engineering, Georgia Institute of Technology and Emory University, Atlanta, Georgia, USA.*
[2]*Department of Cell, Developmental, and Integrative Biology, University of Alabama at Birmingham, Birmingham, AL, USA.*
[3]*Institute for Advanced Biosciences, Centre de Recherche Université Grenoble Alpes, Inserm U 1209, CNRS UMR 5309, Grenoble, France.*
[4]*Department of Chemistry, Emory University, Atlanta, Georgia, USA.*

Table of Contents

Supplementary Table 1: *Oligonucleotide sequences*……………………………………………….4

Supplementary Figure 1: *Chemical structures in oligonucleotide probes*………………………...6

Supplementary Figure 2: *HPLC chromatograms of modified oligonucleotides*………………….....7

Supplementary Figure 3: *MALDI-TOF spectra of oligonucleotides*…………………………….....8

Supplementary Table 2: *Summary of MALDI-TOF results*…………………………………………9

Supplementary Figure 4: *NIH 3T3 fibroblasts form podosomes on fluid RGD-oligonucleotides*..10

Supplementary Figure 5: *Podosome protrusion does not disrupt the SLB*…………………..…11

Supplementary Figure 6: *Conventional tension probes exhibit static quenching*………………12

Supplementary Table 3: *Summary of TCSPC Settings*……………………………………….....12

Supplementary Figure 7: *MT-FLIM probe calibration*……………………………………………..13

Supplementary Figure 8: *MT-FLIM probe density slightly reduces fluorescence lifetime*…….14

Supplementary Figure 9: *Podosome rings and integrin clusters contain β1 integrin*………….15

Supplementary Note 1: *Determination of percentage open probes and local probe density*…17

Supplementary Note 2: *Discussion of MT-FLIM photon statistics*………………………………19

Supplementary Figure 10*: Analysis of MT-FLIM photon statistics*………………………………20

Supplementary Figure 11: *MT-FLIM analysis flowchart*…………………………………..…..21

Supplementary Figure 12: *Invadosomes exert pN integrin tension*……………………………..22

Supplementary Figure 13: *Tension probes unfold specifically under integrin forces*.…………23

Supplementary Figure 14: *MT-FLIM dynamics of podosome maturation*…………………….…24

Supplementary Figure 15: *Analysis of clustering and tension dynamics on static quenched tension probes*…………………………………………………………………………………...26

Supplementary Figure 16: *MFM set-up and excitation-resolved polarization validation*………27

Supplementary Figure 17: *Emission resolved fluorescence data does not indicate lateral organization of integrin forces in podosomes*……………………………………………………..28

Supplementary Figure 18: *Actin polymerization and Rho kinase regulate podosome protrusiveness*……………………………………………………………………...………………29

Supplementary Figure 19: *Myosin IIa is dispensable in podosome force generation on an SLB*…………………………………………………………………………..………………...30

Supplementary Figure 20: *Kinetics of fluorescence recovery after photostimulation*………….32

Supplementary Figure 21: *Integrin tension is released following photocleavable biotin photostimulation*……………………………………………………………………………...…...33

Supplementary Figure 22: *Analysis protocol for photocleavable biotin experiments*………….34

Supplementary Figure 23: *Podosome protrusion is primarily perturbed at the site of PCB photostimulation*………………………………………………………………………………36

Supplementary Figure 24: *NIH-3T3 cell profile*……………………………………………….37

Supplementary References……………………………………………………………………38

Oligo ID	Purpose	Sequence	Source
1	**Hairpin Ligand Strand**	5’-/5Hexynyl TTT GCT GGG CTA CGT GGC GCT CTT /3/AmMO-3’	IDT
2	**4.7 pN Hairpin**	5'-GTG AAA TAC CGC ACA GAT GCG GTA TAA ATG TTT TTT TCA TTT ATA C AAG AGC GCC ACG TAG CCC AGC-3'	IDT
3	**19 pN Hairpin**	5’-GTG AAA TAC CGC ACA GAT GCG CGC CGC GGG CCG GCG CGC GGT TTT CCG CGC GCC GGC CCG CGG CGA AGA GCG CCA CGT AGC CCA GC-3’	IDT
4	**Linear (No Hairpin)**	5'-GTG AAA TAC CGC ACA GAT GCG AAG AGC GCC ACG TAG CCC AGC -3'	IDT
5	**4.7 pN Hairpin (Photocleavable)**	5’-/5PCBio/TT TGT GAA ATA CCG CAC AGA TGC GGT ATA AAT GTT TTT TTC ATT TAT ACA AGA GCG CCA CGT AGC CCA GC-3’	IDT
6	**4.7 Complementary**	5’-GTA TAA ATG AAA AAA ACA TTT ATA C-3’	IDT
7	**19pN Scramble**	5'-GTG AAA TAC CGC ACA GAT GCG TTT ATC GTC AAT ATA TAC GAT ATT TTT TAG AAT CTA GAT GTT AAC TTT TTA AGA GCG CCA CGT AGC CCA GC-3'	IDT
8	**Scramble Complementary**	5’-AAG TTA ACA TCT AGA TTC TAA AAA ATA TCG TAT ATA TTG ACG AT-3’	IDT
9	**MT-FLIM Anchor/Quencher Strand**	5’-CGC ATC TG(I-TBHQ1) GCG GTA TTT CAC TTT/3Bio/-3’	BT
10	**Unlabeled Bottom Strand**	5’-CGC ATC TGT GCG GTA TTT CAC TTT-3’	IDT
11	**MTFM Anchor/Quencher Strand**	5’-/5BHQ1/CGC ATC TGT GCG GTA TTT CAC TTT/3Bio/-3’	BT
12	**MTFM Quencher Strand**	5’-/5BHQ1/CGC ATC TGT GCG GTA TTT CAC TTT-3’	BT
13	**Alkyne-Amine-Biotin**	5’-/Hexynyl/CG CAT CTG TGC GGT ATT TCA C/iAmMC6T/TTT/3Bio/-3’	IDT
14	**TGT Ligand Strand***	5’-/5Hexynyl/GTG AAA TA**G** CGC ACA GAT GCG/3AmMo/-3’	IDT
15	**12 pN TGT Anchor Strand**	5’-/CGC ATC TGT GCG GTA TTT CAC /iAmMC6T/ TT T /3Bio/-3’	IDT
16	**56 pN TGT Anchor Strand**	5’-/5Biosg/TTT/AmMC6T/CGC ATC TGT GCG GTA TTT CAC-3’	IDT
17	**4.7 (TTT) pN Hairpin****	5'-GTG AAA TAC CGC ACA GAT GCG TTT GTA TAA ATG TTT TTT TCA TTT ATA C TTT AAG AGC GCC ACG TAG CCC AGC-3'	IDT
18	**4.7 (TTT) Complementary****	5’-AAA GTA TAA ATG AAA AAA ACA TTT ATA C AAA-3’	IDT

Supplementary Table 1: Oligonucleotide sequences. Summary of oligonucleotides used in this study. Oligo ID is used to denote sequence in **Supplementary Figs. 1,2 and Supplementary Table 2**. IDT = Integrated DNA Technologies, BT = Biosearch Technologies.

* Note: TGT sequences in this work contain a single base pair mismatch (bolded) in the TGT Ligand strand (Oligo 14). Sequences used here are as written, however, for future studies, this mismatch should be corrected: **G→C**. Despite this mismatch, we proceeded with the interpretation of our data. Although to our knowledge, there is no model describing the impact of a mismatch on the tension tolerance, T_{tol} (discussed in **Figure 5**), single molecule force spectroscopy suggests that only the termini of the duplex are critical to defining T_{tol}. According to the deGennes model[1], T_{tol} is sequence independent and follows the equation:

$$T_{tol} = 2f_c\left[\chi^{-1}tanh\left(\chi\frac{L}{2}\right) + 1\right] \quad (1)$$

$2f_c$ is the tension tolerance per bond, L is the sequence length (bp), and χ^{-1} is the number of force-bearing base-pairs at the end of a duplex. Measuring the T_{tol} of DNA duplexes using magnetic tweezers revealed that χ^{-1} equals 6.8 base-pairs[2]. Therefore, although a mismatch at bp 9 will slightly reduce the duplex's thermal stability, it will not significantly impact T_{tol}.

**Note: These oligos were used only in absorbance spectroscopy of static quenched probes, in accordance with Zhang, et. al[3]. All other experiments using 4.7 pN tension probes used Oligo 2.

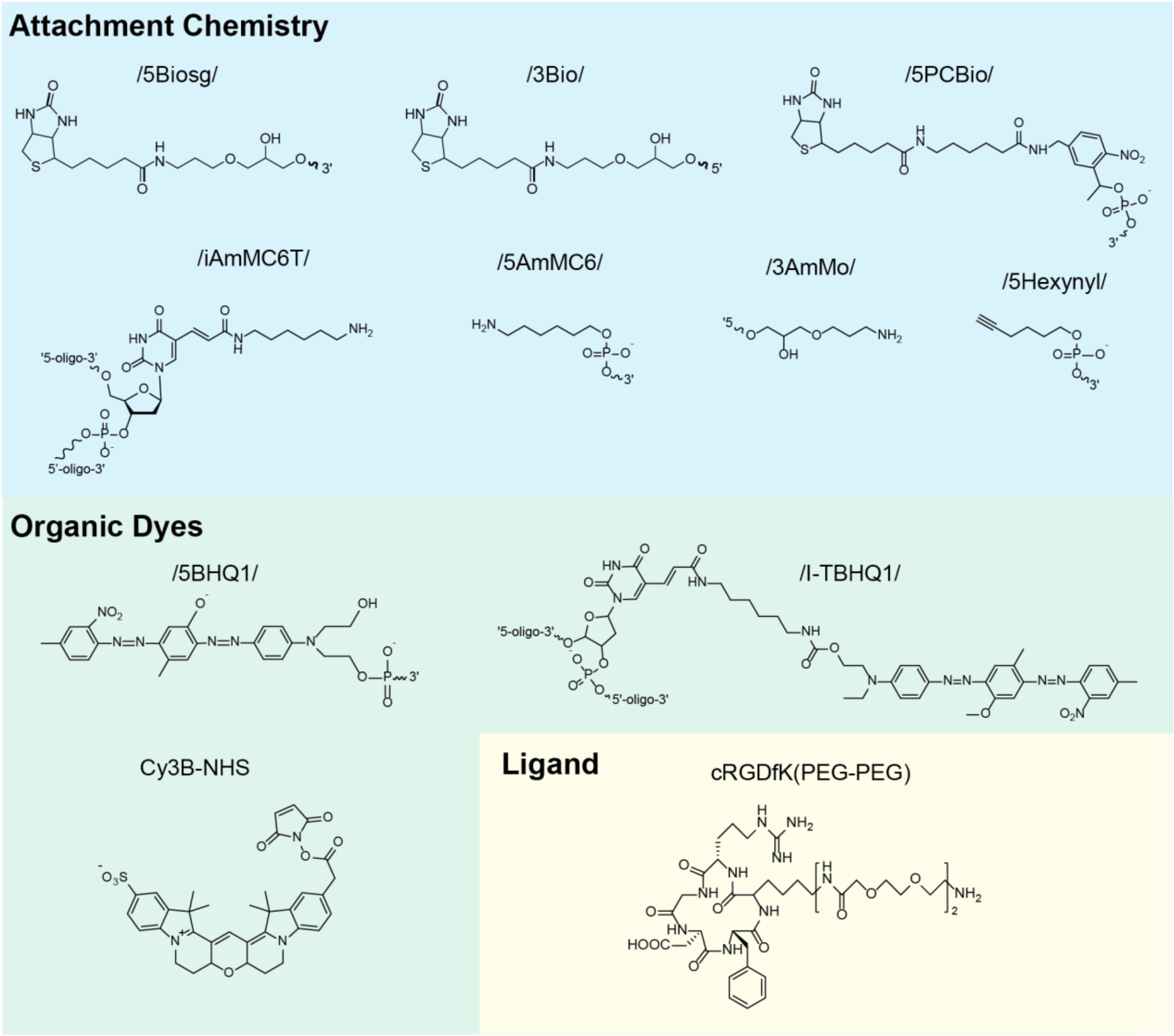

Supplementary Figure 1: Chemical structures in oligonucleotide probes. Chemical structures for relevant DNA modifications. Cy3B and cRGDfK(Peg-PEG) were conjugated as described.

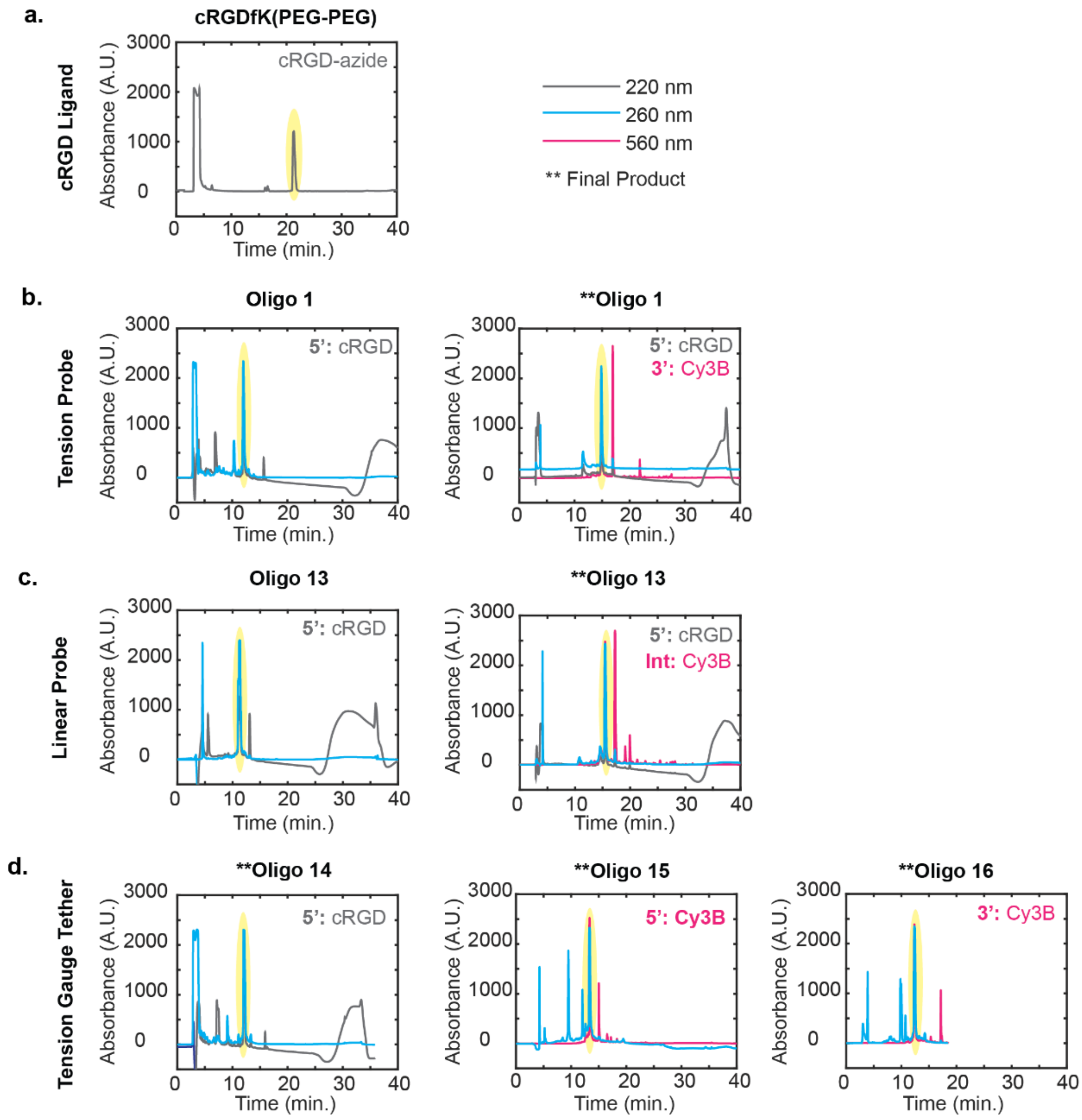

Supplementary Figure 2: HPLC chromatograms of modified oligonucleotides. (a-d) Chromatograms of cRGD and DNA probes. Oligos sequences are as shown in Supplementary Table 1. Grey, blue, and pink lines are for cRGD, DNA, and Cy3B, respectively. Product peaks are highlighted in yellow, and HPLC spectra for final products are marked**. Final products were validated by MALDI-TOF (Supplementary Fig. 3 Supplementary Table 2). The locations of the cRGD and Cy3B are indicated as 5', 3', or Int for internal modifications.

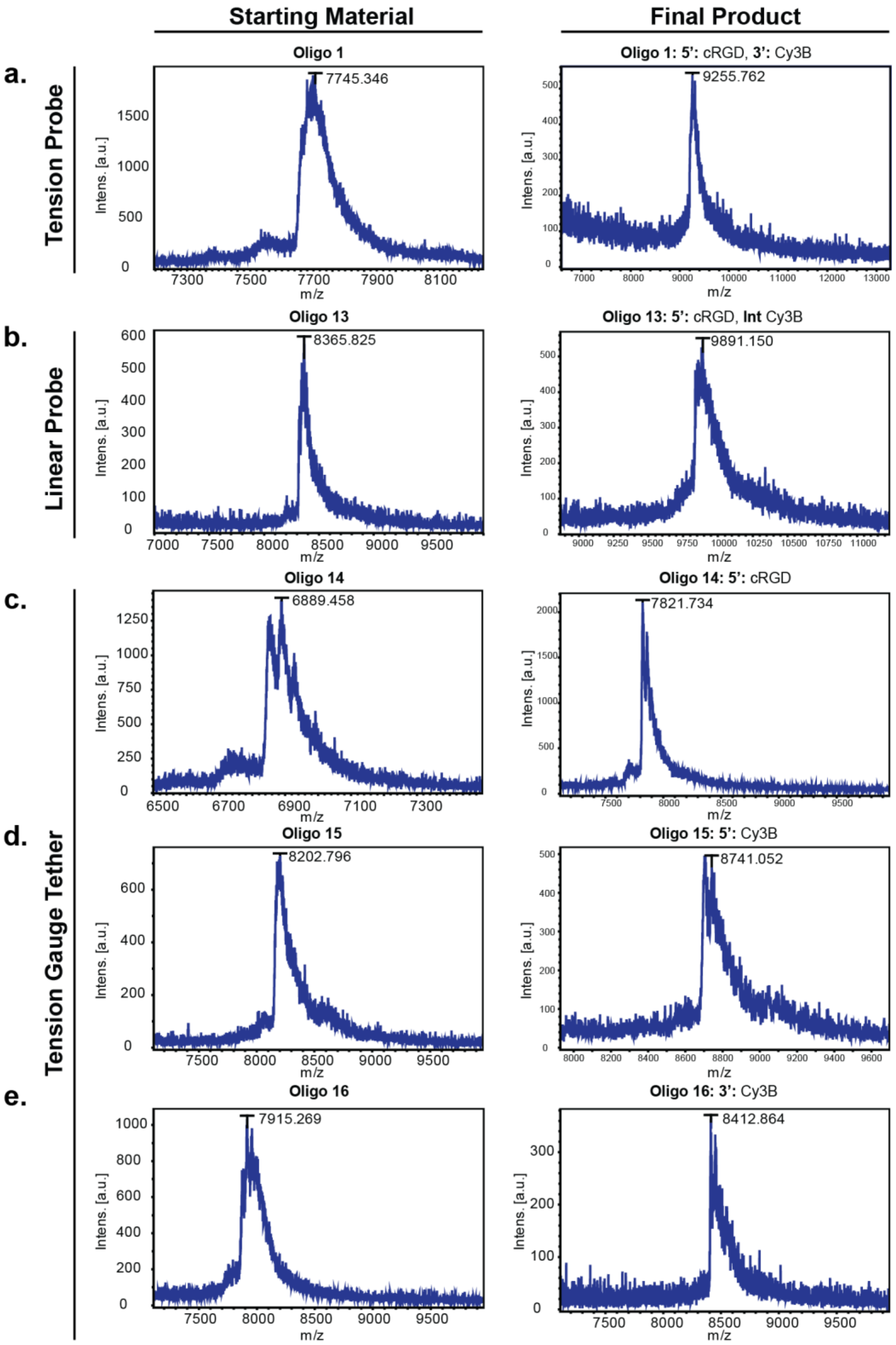

Supplementary Figure 3: MALDI-TOF spectra of oligonucleotides (a-e) Mass spectra of starting materials and final probe products.

MALDI	Sequence	Starting or Product	Calculated MW	Measured MW	Difference (%)
**a.**	**1**	Starting	7729.0	7745.346	0.211
		Product	9361.96	9255.762	-1.134
**b.**	**13**	Starting	8356.7	8357.821	0.001
		Product	9989.66	9891.15	-0.986
**c.**	**14**	Starting	6875.5	6889.458	0.203
		Product	7852.6	7821.734	-0.392
**d.**	**15**	Starting	6875.5	6889.458	0.203
		Product	7852.6	7821.734	-0.392
**e.**	**16**	Starting	7903.3	7915.269	0.151
		Product	8559.21	8412.164	-1.718

Supplementary Table 2: *Summary of MALDI-TOF results.*

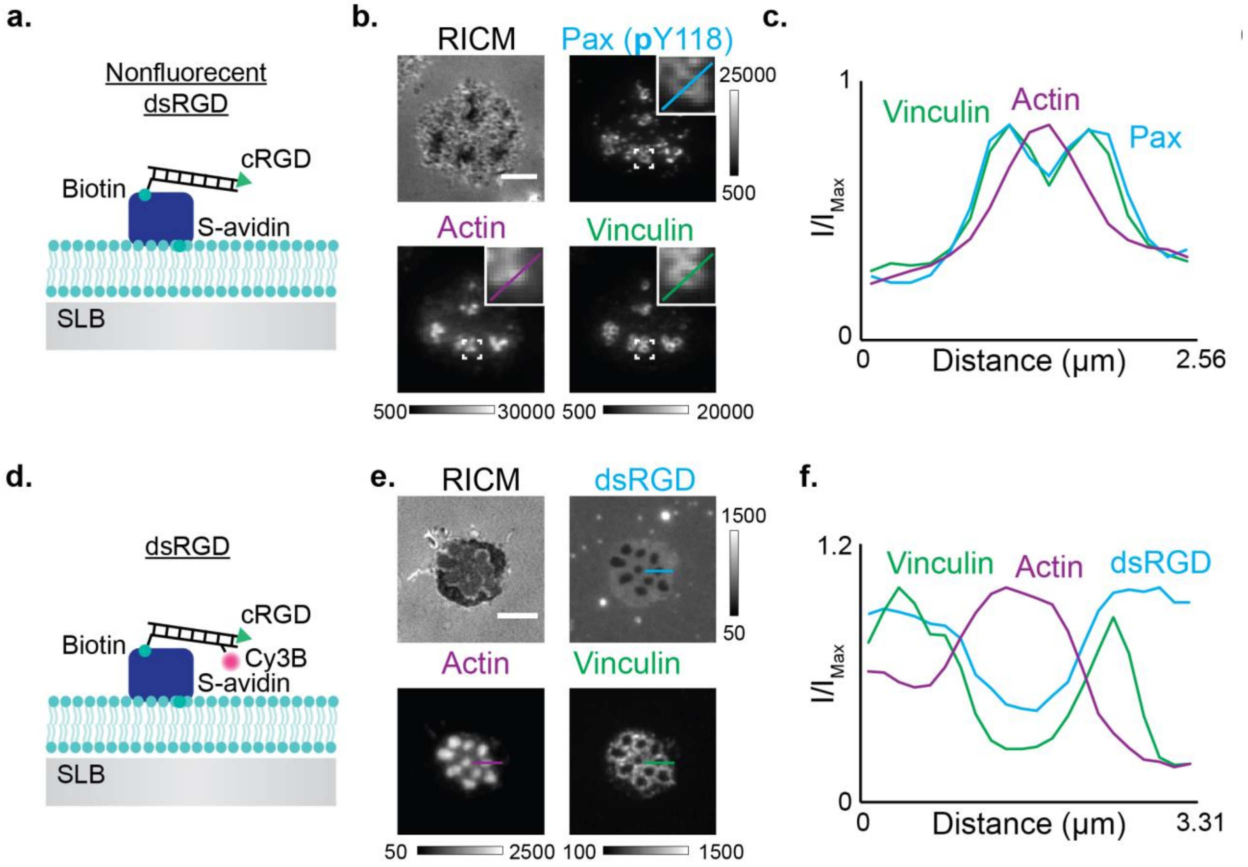

Supplementary Figure 4*:* NIH 3T3 fibroblasts form podosomes on fluid RGD-oligonucleotides. (a) Schematic of unlabeled cRGD-functionalized DNA probes tethered to an SLB. **(b)** Immunostaining of podosome-forming NIH-3T3 cell stained with Phospho-Paxillin Tyr 118 Polyclonal Antibody, Phalloidin-iFluor 488, and Vinculin Antibody SF647. (N=85 cells, 3 experiments.) **(c)** Normalized linescan analysis of podosome in **b.** Blue, green, and purple lines represent **p**Y118, vinculin, and actin, respectively. **(d)** Schematic of fluorescently-tagged DNA probes presenting cRGD ligands on an SLB. **(e)** Immunostaining of podosome-forming NIH 3T3 cells (n=97, 4 experiments). Cells were stained with Phalloidin-iFluor 488 and Vinculin Antibody SF 647. **(f)** Normalized linescan analysis of podosomes in **b.** Blue, green, and purple lines represent dsRGD, vinculin, and actin, respectively.

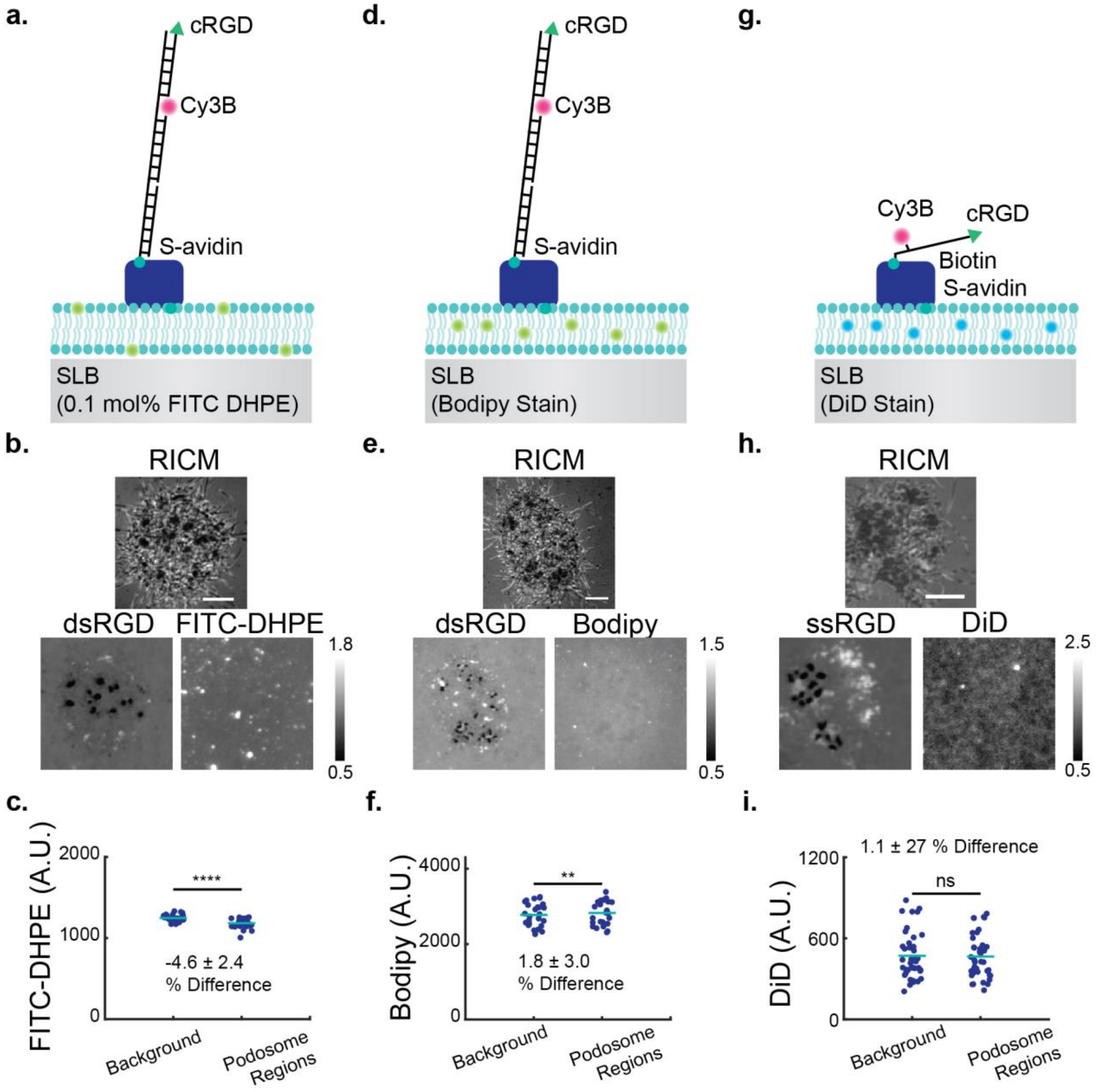

Supplementary Figure 5: Podosome protrusion does not disrupt the SLB. (a,d,g) Schematic of oligonucleotide probes and membrane labels used in **b** and **c**, **e** and **f**, and **h** and **i**, respectively. **(b,e,h)** Representative epifluorescence images of podosomal depletion as indicated by Cy3B-DNA depletion. The SLB beneath was labeled with FITC-labeled lipids or stained with membrane intercalating dyes, as indicated. Fluorescence images are displayed normalized to the SLB background. **(c,f,i)** Average membrane fluorescence beneath podosome depletion regions versus the SLB background. Statistics were performed with a two-tailed Wilcoxon matched-pairs signed rank test (**c,f**) or a two-tailed, paired Students t-test (**i**). Teal bars represent the median (**c,f**) and the mean (**i**). P>0.05, **P<0.01, ****P<0.0001. Statistics were calculated on at least 26 cells, 3 experiments. Outliers were excluded (median ± 3 scaled median absolute deviations). Source data are provided as a Source Data file.

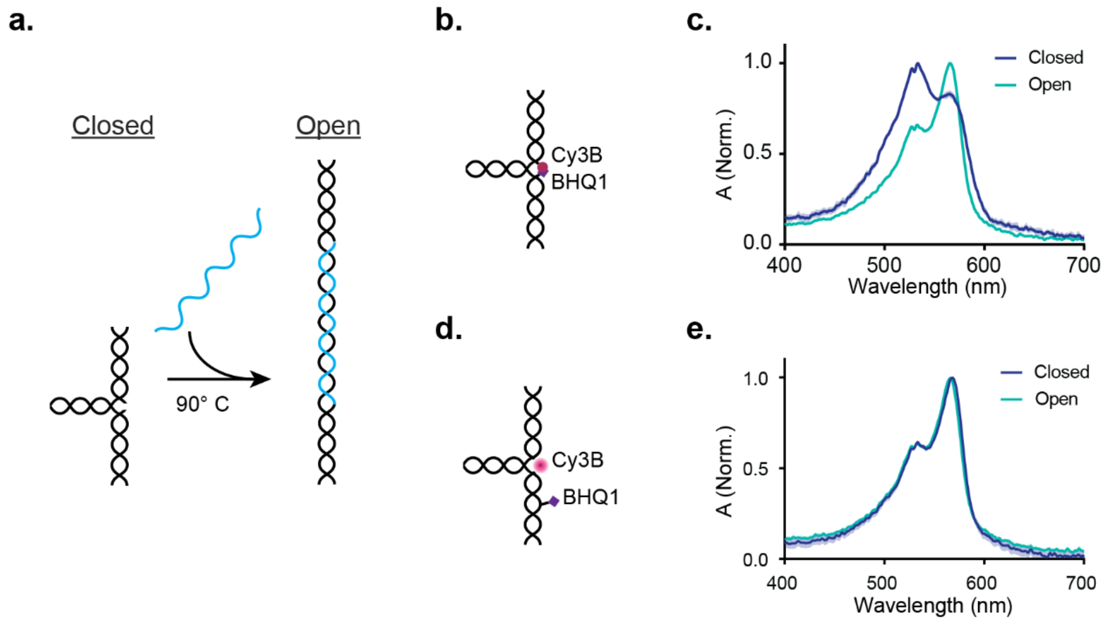

Supplementary Figure 6: Conventional tension probes exhibit static quenching. (a) Tension probes were chemically unfolded by annealing with a strand complimentary to the stem-loop sequence (blue). **(b,d)** Schematic of conventional and MT-FLIM probes, respectively, in solution. **(c,e)** Absorbance spectra of closed (dark blue lines) and open (teal lines) conventional and MT-FLIM probes, respectively. Conventional MTFM probes exhibited a shift indicative of static quenching. Thus, these probes are referred to as static quenched probes throughout the manuscript. Data represent the mean ± s.e.m. N = 3 experiments. Source data are provided as a Source Data file.

Supplementary Table 3: Summary of TCSPC Settings

**General**	
TCSPC Resolution	25.0 ps
TCSPC Mode	T3
Time-Trace Binning	30 ms
**Sync**	
CFD Level	-150 mV
Zero Cross	-10 mV
Sync Divider	8
**Detector**	
CFD Level	-45 mV
Zero Cross	-10 mV
Offset	530 ps

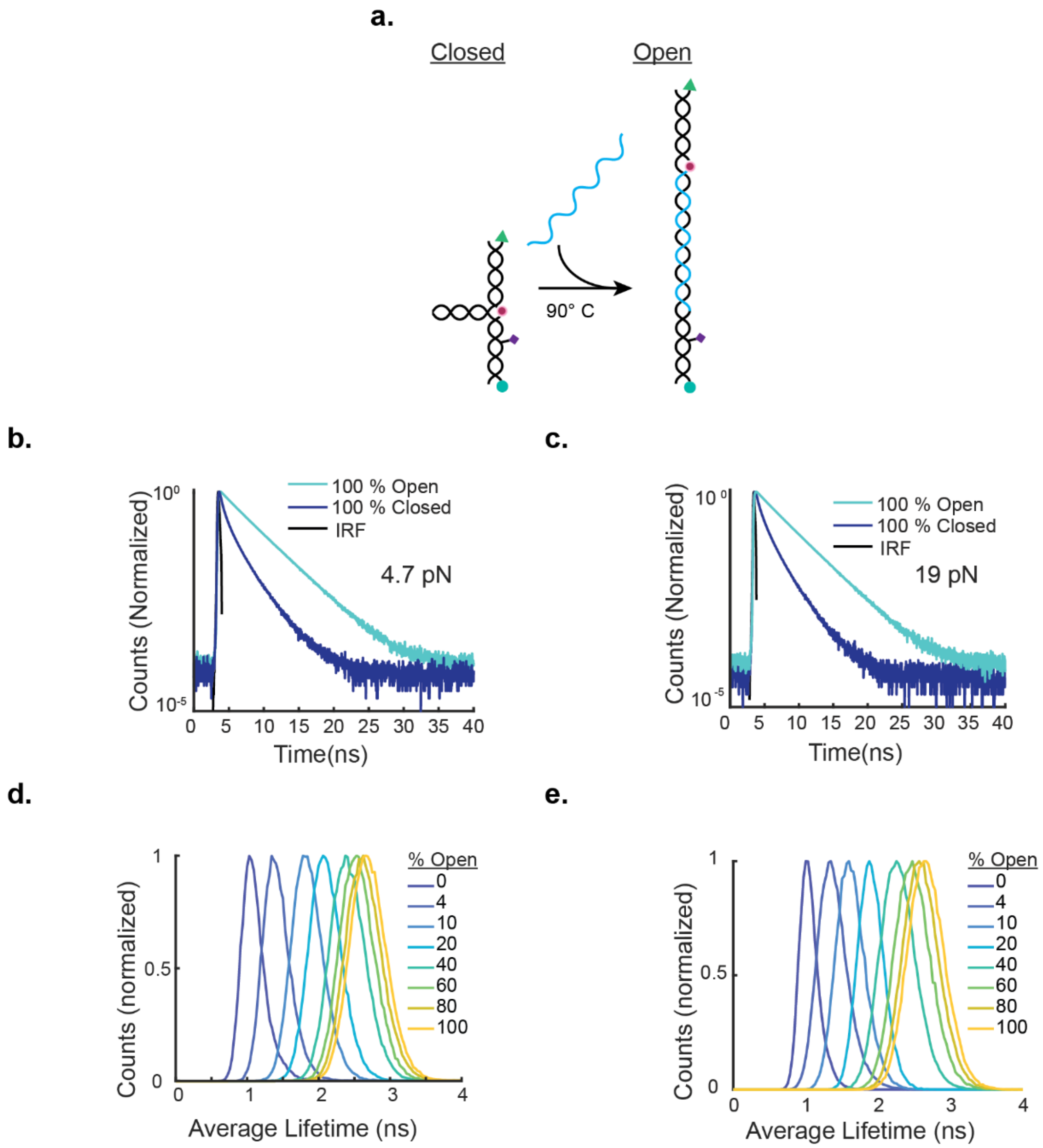

Supplementary Figure 7: MT-FLIM probe calibration. (a) To open probes, hairpins were thermally annealed to a sequence complimentary to the stem-loop. **(b,c)** Representative fluorescence decay histograms for opened and closed 4.7 and 19 pN tension probes, respectively. Closed probes exhibit a multiexponential decay (3 experiments). The instrument response function (IRF, calculated in software) is shown in black. Photon counts are displayed on a logarithmic scale. **(d,e)** Representative average fluorescence lifetime histograms for SLBs with increasing percent open 4.7 and 19 pN tension probes, respectively (2-3 experiments per condition). Here, counts refers to the number of pixels with a given average lifetime.

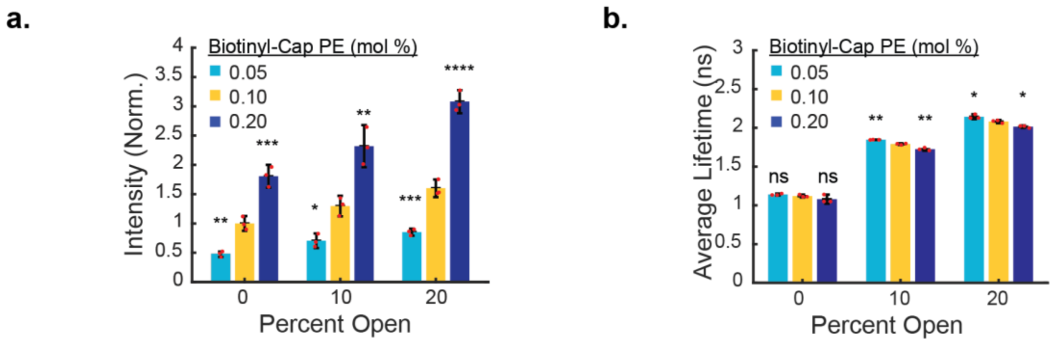

Supplementary Figure 8: MT-FLIM probe density slightly reduces fluorescence lifetime. **(a)** Normalized fluorescence intensity of SLBs containing 0-20% open probes on SLBs with 0.05 – 0.2 mol% Biotinyl-Cap PE. **(b)** Average fluorescence lifetime on SLBs containing 0-20 % open probes on SLBs with 0.05-0.2 mol% Biotinyl-Cap PE. Light blue, yellow, and dark blue bars represent 0.05, 0.1, and 0.2 mol% Biotinyl-Cap PE, respectively. Red circles represent the means of individual experiments. Data points represent the mean of individual experiments. Bars bars represent the mean ± s.e.m (error bars), 3 experiments. Statistics were performed with an ANOVA followed by a multicomparison test comparing each data point to the corresponding 0.1 mol% Biotinyl-Cap PE data (yellow bars). ns P>0.05, *P<0.05, **P<0.01, ****P<0.0001. Source data are provided as a Source Data file.

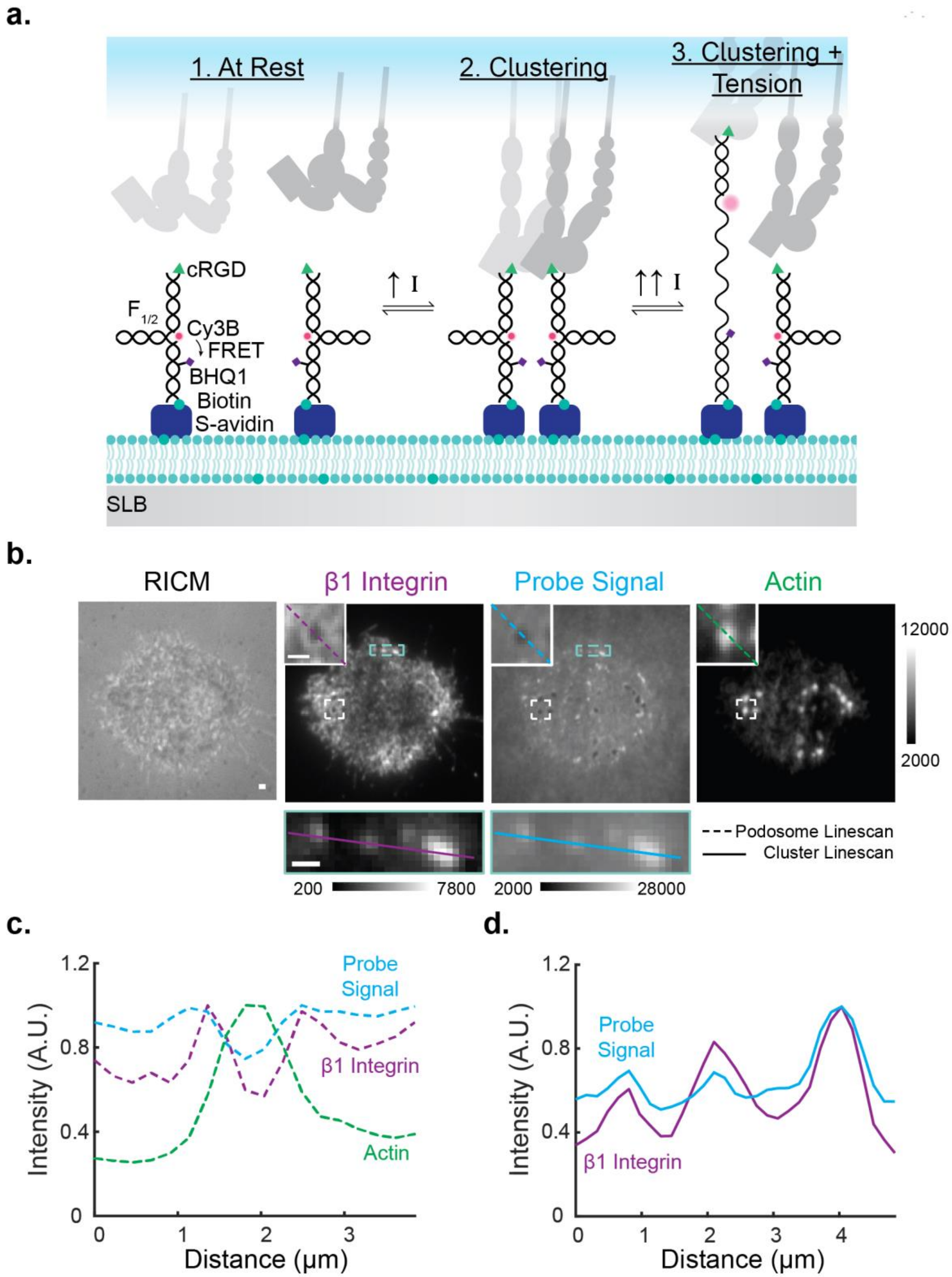

Supplementary Figure 9: Podosome rings and integrin clusters contain β1 integrin. (a) MT-FLIM probes were used with intensity-based imaging, such that both clustering and opening events could cause an increase in fluorescence signal. **(b)** Representative images of podosome-forming cells show colocalization of β1 integrins with RGD clusters (solid lines) and

podosome rings (dashed lines) in the probe channel. β1 integrins and actin were detected by Anti-Integrin β1 Antibody, clone MB1.2 followed by Alexa Fluor 647 goat anti-mouse IgG_{2b} (γ2b) and Alexa 488- Phalloidin, respectively. **(c,d)** Linescan analysis of podosomes **(c)** and clusters **(d)** shows good colocalization of β1 integrin and probe signal. Linescan locations are indicated in **b**. Green, blue, and purple lines represent actin, probe signal, and β1 integrin, respectively. N = 16 cells, 2 experiments. Scale Bars, 5 μm.

Supplementary Note 1: Determination and interpretation of percentage open probes and local probe density

The intensity of tension probe signal, I, measured as the number of photon counts per pixel, is a function of the local probe density, ρ, the fraction of open and closed probes, O and C, their per-probe photon count contributions, m and n, and the dark counts, D.

$$I(O, C, \rho) = \rho\big(m(O) + n(C)\big) + D \tag{2}$$

Because probe opening is binary (probes are either open or closed), O and C are related such that:

$$O + C = 1 \tag{3}$$

Assuming that in the SLB background, all probes are closed and the relative probe density is 1, we find that:

$$I(0,1,1) = n + D = I_0 \tag{4}$$

Although m and n cannot be measured directly, they are related by the quenching efficiency, QE such that:

$$QE = \frac{m-n}{m} \tag{5}$$

$$m = \frac{n}{1-QE} \tag{6}$$

Rearranging (2) and substituting with (3)-(6), we find that probe density,

$$\rho = \frac{I(O,C,\rho)-D}{\left(\frac{I_0-D}{1-QE}(O)+(I_0-D)(1-O)\right)} \tag{7}$$

We obtained QE from the slope of the linear curve-fit epifluorescence images of titrated open and closed probes (Figure 2c). To determine D, we calculated the average number of photons per pixel on the second detector blocked with a 690 nm band-pass filter during calibration image acquisition. Since the average number of photons was less than 1 per pixel, we determined dark counts to be negligible. To determine O, the fraction of open probes per pixel, $F(\tau)$, we used a 5%-open-interval look-up-table (LUT) generated from the empirical calibration curve of percent open probes versus average fluorescence lifetime per pixel, τ, (Figure 2b, Supplementary Figure 7d,e), which fit to the biexponential equation:

$$\tau = ae^{bO} - ce^{dO} \tag{8}$$

Here τ is the mean photon arrival time determined by the Fast FLIM algorithm in SymPhoTime 64. This metric provides the distance between the rise of the IRF and the center-of-mass of photon arrivals in a decay and does not require curve-fitting. This method has some disadvantages such as uncertainty due to uncertainty of time-zero and contribution of background photons; therefore, it is not typically the most quantitative metric to characterize a system and is typically used to give an initial real-time FLIM image. However, it has still proven useful as an estimate of the fluorescence lifetime in a variety of FLIM applications[4, 5, 6]. For our purposes, we found it to be a robust measure of the fluorescence lifetime of our tension-probe surfaces, in which empirically measured lifetimes were much more important than the physical processes giving rise to these exact lifetimes. When used with our calibration curve to convert

the average fluorescence lifetime per pixel to the percentage of open probes per pixel, the precision is related to the number of photons in the calibration curve image, which we held at constant of approximately 10^5 photons in the peak of the fluorescence lifetime decay curve, as well as with the number of photon counts in the pixel of interest. The major sources of uncertainty in this metric comes from the width of the histogram used to generate the percentage of open probes look-up-table and uncertainty due to the computed IRF. Because we were concerned that dwell time could cause some probe melting and affect the fluorescence lifetime, we performed these measurements for all imaging conditions (0.14 and 0.08), but this effect was negligible. The constants $a - d$ were as follows:

**Probe (pN)**	**a**	**b**	**c**	**d**	**r^2**
4.7	2.306	0.001	-1.166	-0.072	0.9996
19	2.295	0.002	-1.21	-0.056	0.9992

While we found the average fluorescence lifetime of probes on our SLBs to be consistent, it was important to minimize the free dye in solution, which could shift the lifetimes. We recommend that anyone using this method generate a calibration curve on their own instrument prior to data quantification.

Supplementary Note 2: Discussion of MT-FLIM photon statistics.

A challenge in MT-FLIM interpretation was determining the cutoff between signal and noise. In our raw average fluorescence lifetime images, we observed an increase in fluorescence lifetime even in regions inside the podosome core depletion zone, where integrin receptors are excluded[7]. In very static podosomes in MT-FLIM (60x objective, 1 min acquisition) and on conventional tension probes (100x objective, 500 ms exposure time) (**Supplementary Fig. 15c,e**), it was apparent that signal was contributed primarily by the adhesion ring. We attributed this effect to three factors: (1) Podosomes are micron-sized structures and are subject to the diffraction limit. Thus, high fluorescence lifetime photons from the ring could be collected in depletion regions. (2) Podosomes are dynamic structures. Any movement of the ring could cause slight blurring of signal. (3) Depletion regions have low signal-to-noise ratio (SNR) and are subject to influence by noise (detector shot noise and otherwise). We hypothesize that these three features together give rise to the appearance of tension in the podosome core. Considering a primarily depleted (dark) podosome core, some photon contribution from the ring will increase the photon count, but it will still be much darker than the surrounding regions. However, assuming that most of these photons have a long fluorescence lifetime, the it will appear that the core region has a high percentage of open probes. This effect would be magnified by any movement or changes of the podosome ring or core structure during imaging.

To determine which pixels were the most reliable, we performed an extensive analysis of MT-FLIM photon statistics. It is desirable to maximize photon counts, because the SNR equals $\sqrt{n}$, where n is the number of photons. Since each photon in a FLIM image is time-tagged, FLIM images can be subdivided into different frame widths. Images in Fig. 2 were collected for 3 min, but we empirically selected an acquisition time of 60 s for analysis as a compromise between podosome dynamics and the need for a maximal photon count. To demonstrate this effect, we have subdivided a podosome image acquisition into time-bins (Supplementary Fig. 10a,b). To determine the tolerable minimum SNR, or photon count, we analyzed τ versus photons counts per pixel. From these data, we observed that some low photon pixels contributed long fluorescence lifetimes that were inconsistent with Cy3B's fluorescence lifetime (data not shown). Using our calibration curves, we defined the maximum reasonable fluorescence lifetime per pixel to be 2.97 ± 0.04 ns (mean ± s.e.m., 6 surfaces), which corresponds to one standard deviation above the average fluorescence lifetime of an SLB containing 100% open probes. Note that here the standard deviation describes the width of the histogram data rather than the variation of mean lifetime across experiments. 19 and 4.7 pN surfaces did not have a statistically significantly different fluorescence lifetime for open probes, so this 2.97 ± 0.04 ns lifetime applied to both data sets. To determine a photon count cutoff, we identified regions associated with the SLB background, clusters, and podosomes and plotted histograms of fluorescence lifetime and photon counts per pixel for each of our hairpins (**Supplementary Fig. 10c,d**). Since the average number of photons per pixel with $\tau > 2.97$ in podosomes was 23 ± 12 photons (mean ± s.d.), we set the minimum SNR tolerance to 1:5. Therefore, in all MT-FLIM analysis, only pixels with $\tau < 2.97$ ns and photon counts > 25 were considered.

Mean Photon Count per Pix. with τ > 2.97 ns

	**Linear**	**4.7 pN**	**19 pN**
**SLB Background**	n/a	73 ± 14	n/a
**Clusters**	n/a	n/a	n/a
**Podosomes**	16 ± 6	30 ± 18	22 ± 11

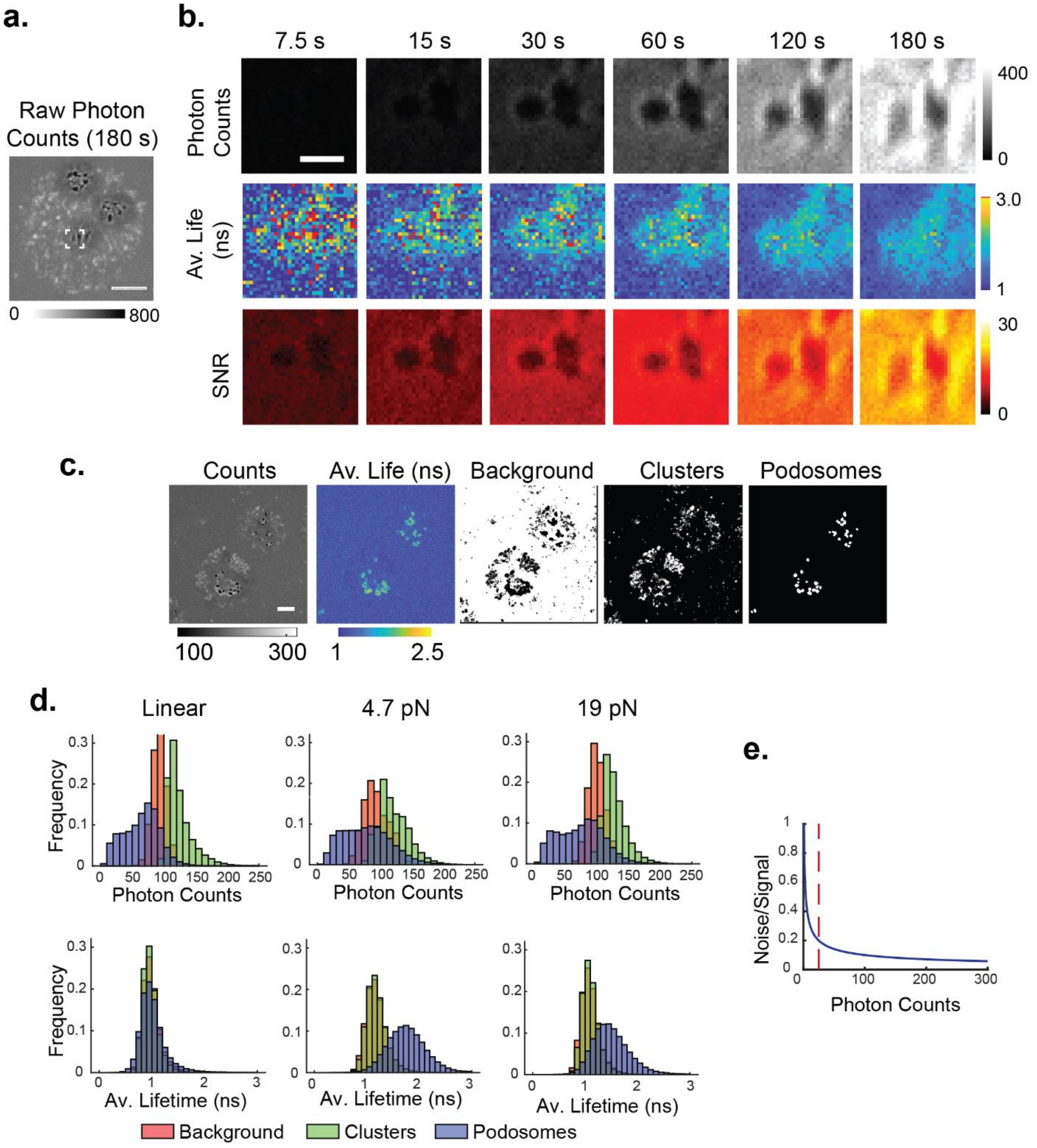

Supplementary Figure 10*:* **Analysis of MT-FLIM photon statistics. (a)** Representative 3-minute photon counts image of podosomes on 4.7 MT-FLIM probes. Scale bar, 5 µm. **(b)** Podosome zoom-in of white box in **a** subdivided into 7.5 - 180 s bins. For MT-FLIM analysis, 60 s image bins were used to maximize SNR while minimizing temporal blurring. Scale bar, 1 µm. **(c)** Representative 4.7 pN MT-FLIM image and maps of the background, clusters, and podosomes. Features were identified by size and intensity-based-thresholding. Scale bar, 5 µm. **(d)** Histogram analysis of the average photon counts and fluorescence lifetime per pixel on MT-FLIM probes. Red, green, and blue histograms represent background, cluster, and podosome

photons, respectively. (N = 48 Images, 3 experiments). **(e)** Plot showing the noise-to-signal ratio versus photon counts. The red dashed line represents the 25-photon cutoff. Source data are provided as a .mat Source Data file.

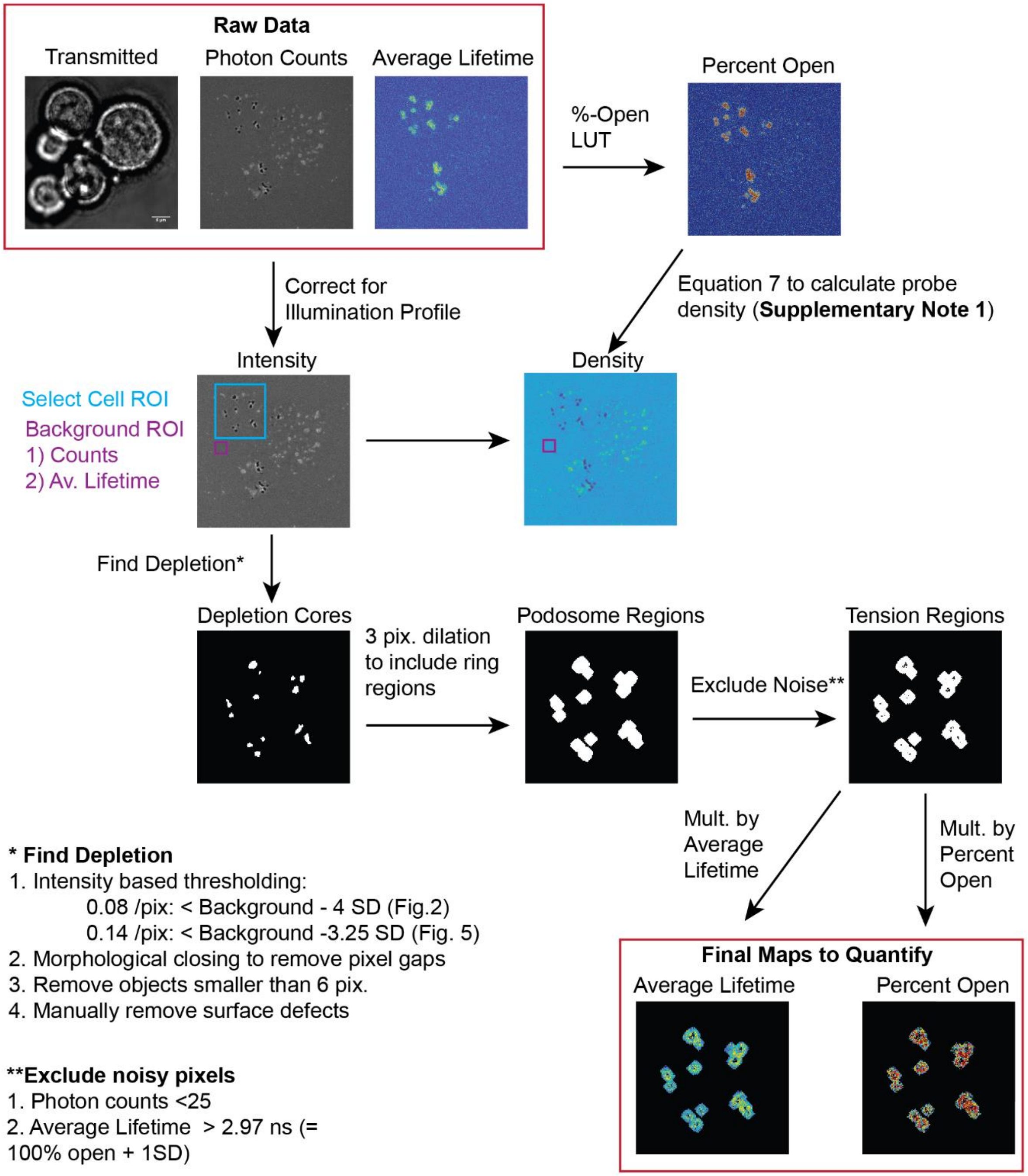

Supplementary Figure 11: MT-FLIM Analysis Flowchart. MT-FLIM images were read into MatLab using the BioFormats plugin. To account for the uneven confocal illumination profile, the raw photon counts data was corrected using an average illumination profile generated from SLBs lacking cells. For each cell, we selected an ROI containing the cell and a local background

ROI, which was used to define the background photon counts and fluorescence lifetime. To identify podosomes, we first identified podosome cores as described in steps 1-4, above (*). Surface defects, such as holes in the SLB, were manually removed. To include the podosome ring region, we performed a 3-pixel dilation on podosome cores. Noisy pixels as determined in Supplementary Note 2 were excluded in the final masks (**). To determine the percentage of open probes and the relative probe density, we applied our empirical look-up table and Equation 7, which are described in detail and derived in Supplementary Note 1. Percent open and average fluorescence lifetime maps were multiplied by the final podosome mask, and statistics were performed on a per cell-level, unless otherwise stated. Scale bar, 5 μm.

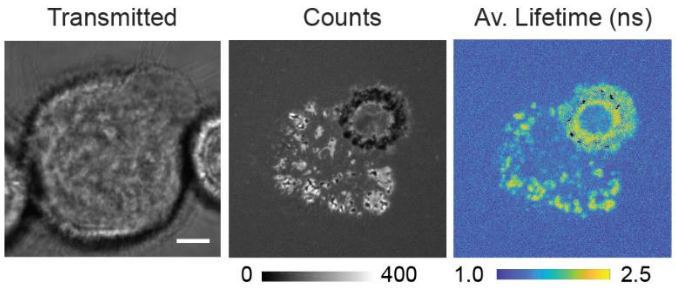

Supplementary Figure 12: Invadosomes exert pN tension. Myf cells were seeded on SLBs presenting 4.7 pN (shown here) or 19 pN tension probes and imaged following ~1 hour of cell spreading. Image depicts a full three-minute acquisition. Scale Bar, 5 μm. (N = 61 cells, 3 experiments).

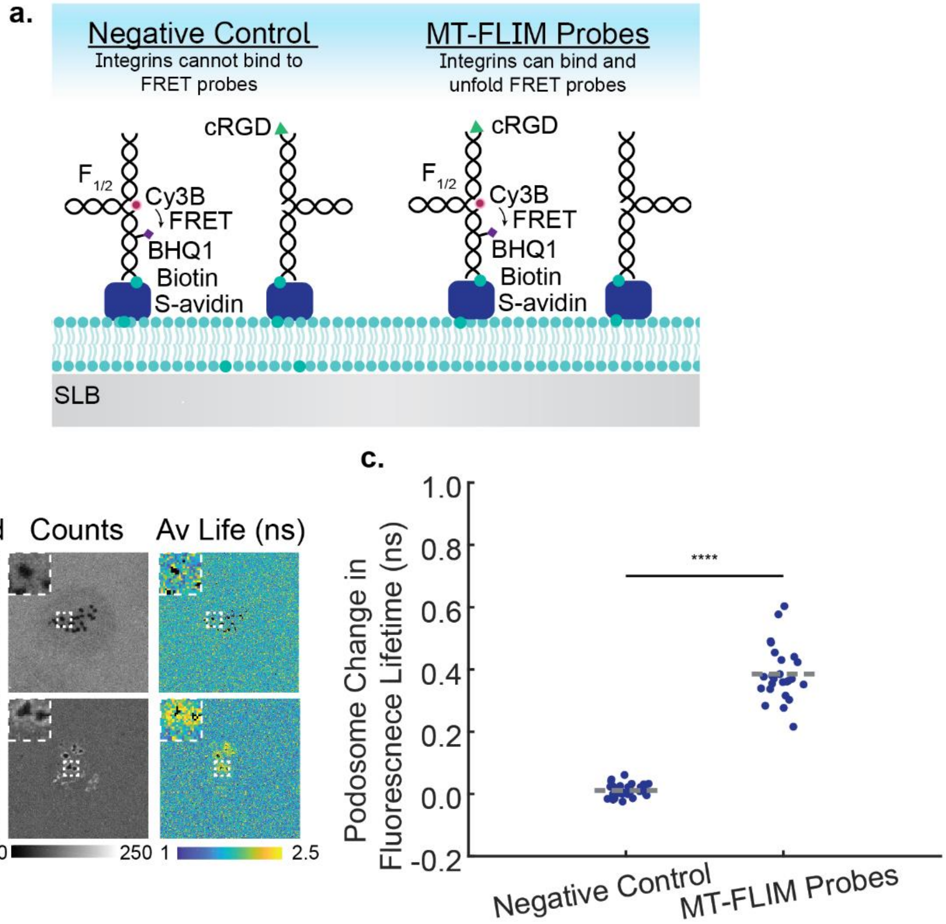

Supplementary Figure 13: Tension probes unfold specifically under integrin forces. (a) DNA probes were co-presented on an SLB such that the ligand and FRET pair were either separated or presented on the same probe. To maintain ligand density, SLBs contained 0.2% mol biotinyl cap PE. **(b)** Representative images of podosomes on co-presented DNA probes. Note that the fluorescence lifetime was higher than in other experiments, likely due to probe co-presentation. Scale bar, 5 µm. **(c)** Average change in fluorescence lifetime in podosome-regions per cell. Dashed grey lines represent the mean. Statistics were performed with a two-tailed paired Students t-test. Each group contained at least 25 cells, 3 experiments. ****$P<0.0001$. Source data are provided as a Source Data file.

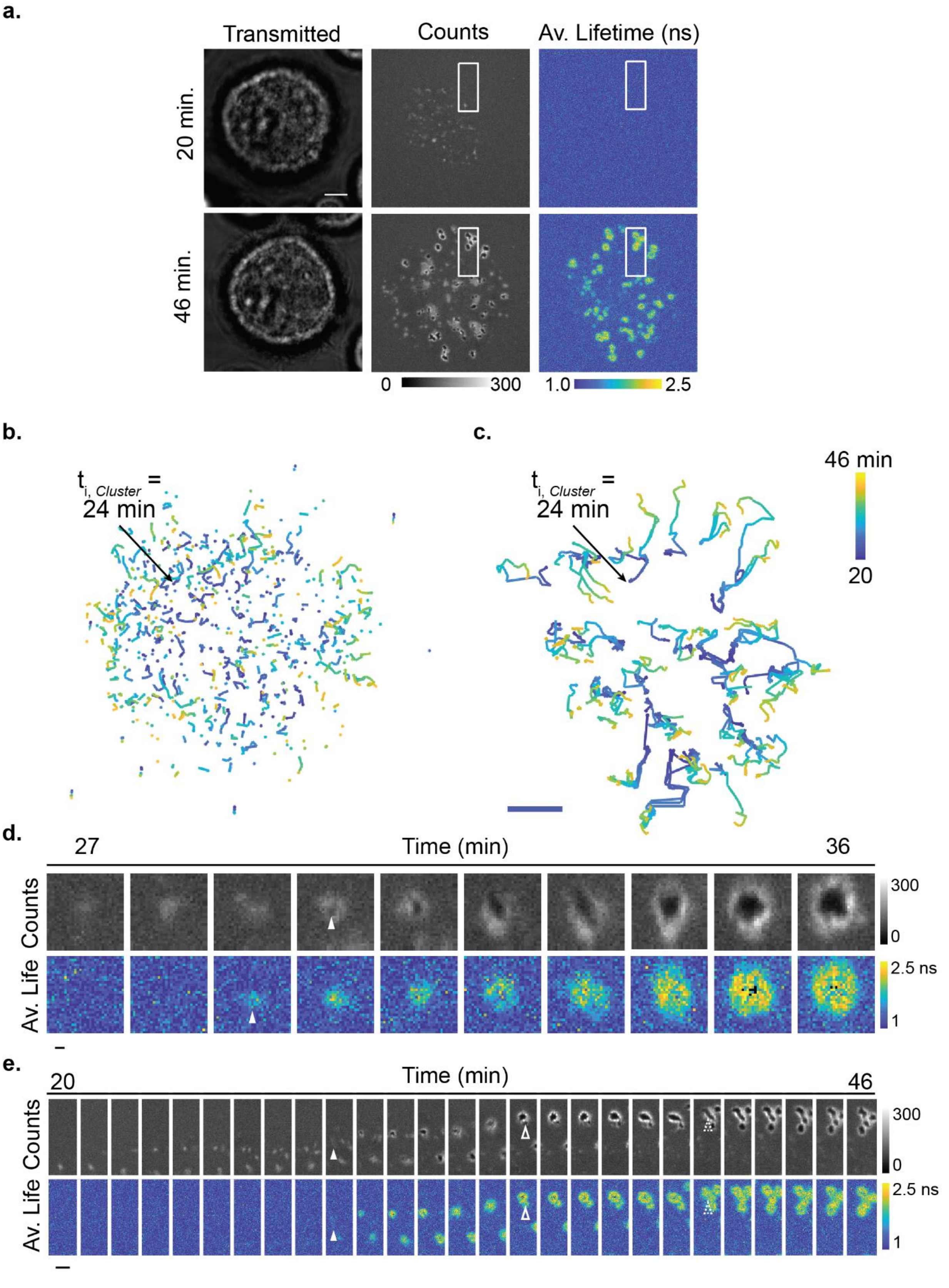
a.
Transmitted
Counts
Av. Lifetime (ns)
20 min.
46 min.
0
300
1.0
2.5
b.
$t_{i, Cluster}$ =
24 min
c.
$t_{i, Cluster}$ =
24 min
46 min
20
d.
27
Time (min)
36
Counts
Av. Life
300
0
2.5 ns
1
e.
20
Time (min)
46
Counts
Av. Life
300
0
2.5 ns
1

Supplementary Figure 14: MT-FLIM dynamics of podosome maturation. (a) MT-FLIM image of the NIH 3T3 cell from **Fig. 2g** at 20 and 46 minutes (N=6 cells, 3 experiments). Scale bar, 5 μm. **(b,c)** Cluster and podosome trajectories from 20 to 46 min. Filled circles represent the emergence of a new cluster (**b**) or podosome/ podosome-preceding cluster (**c**). Scale bar, 5 μm **(d)** Representative kymograph of podosome formation with each frame centered on the centroid of the podosome or cluster noted with the arrow in **b.** White triangles indicate the emergence of tension and depletion. Select frames are reproduced in Fig. 2g. Scale bar, 0.3 μm. **(e)** Kymograph of the white boxed region in **a** showing the emergence and maturation of podosomes from clusters (filled white arrows). Podosomes spread outwards over several minutes, and new podosomes emerge by splitting (dotted white arrows) and *de novo* formation (unfilled white arrows). Scale bar, 2 μm.

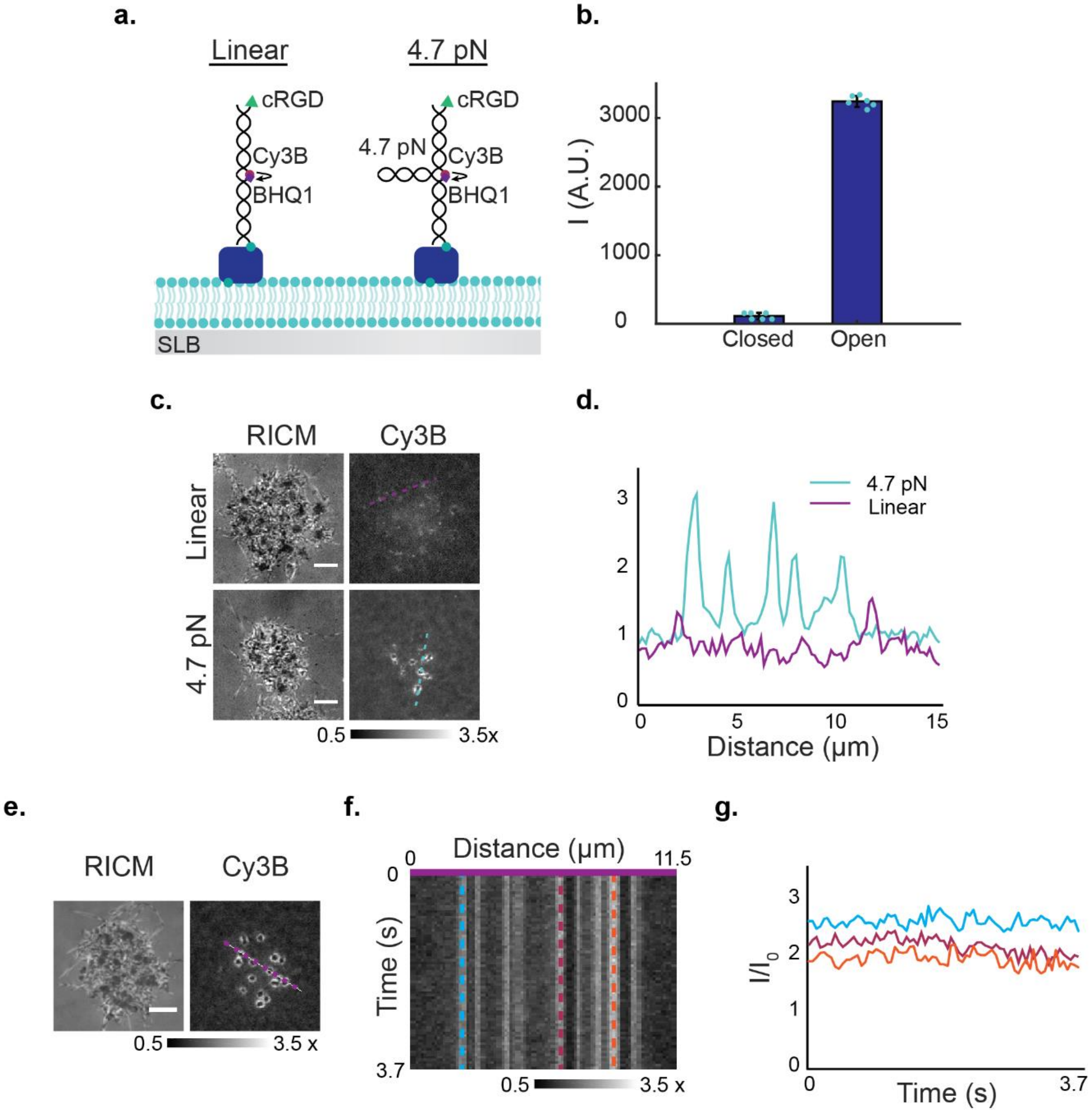

Supplementary Figure 15: Analysis of clustering and tension dynamics on static quenched tension probes. (a) Schematic of conventional MTFM probes with a linear scaffold and 4.7 pN hairpin. **(b)** Determination of MTFM probe quenching efficiency on an SLB. Teal circles represent the mean intensity from 6 individual measurements. Bars represent the mean ± s.d. (error bars), 2 experiments. **(c,d)** Representative images and linescan analysis of NIH 3T3 cells forming podosomes one hour after addition to MTFM probes on an SLB. To minimize the contribution of autofluorescence, Cy3B images are normalized to the non-podosome signal underneath the cell. (N = 42 cells, 3 experiments) **(e)** Representative first frame of a 3.7 s MTFM epifluorescence timelapse with 73 frames to mimic an MFM experiment. **(f)** Kymograph of linescan in **(e)**. **(g)** Linescan analysis of 3 representative podosomes in **f** show only small noise-dominated fluctuations in MTFM ring signal (N = 35 cells, 3 experiments). All scale bars, 5 μm. Source data are provided as a Source Data file.

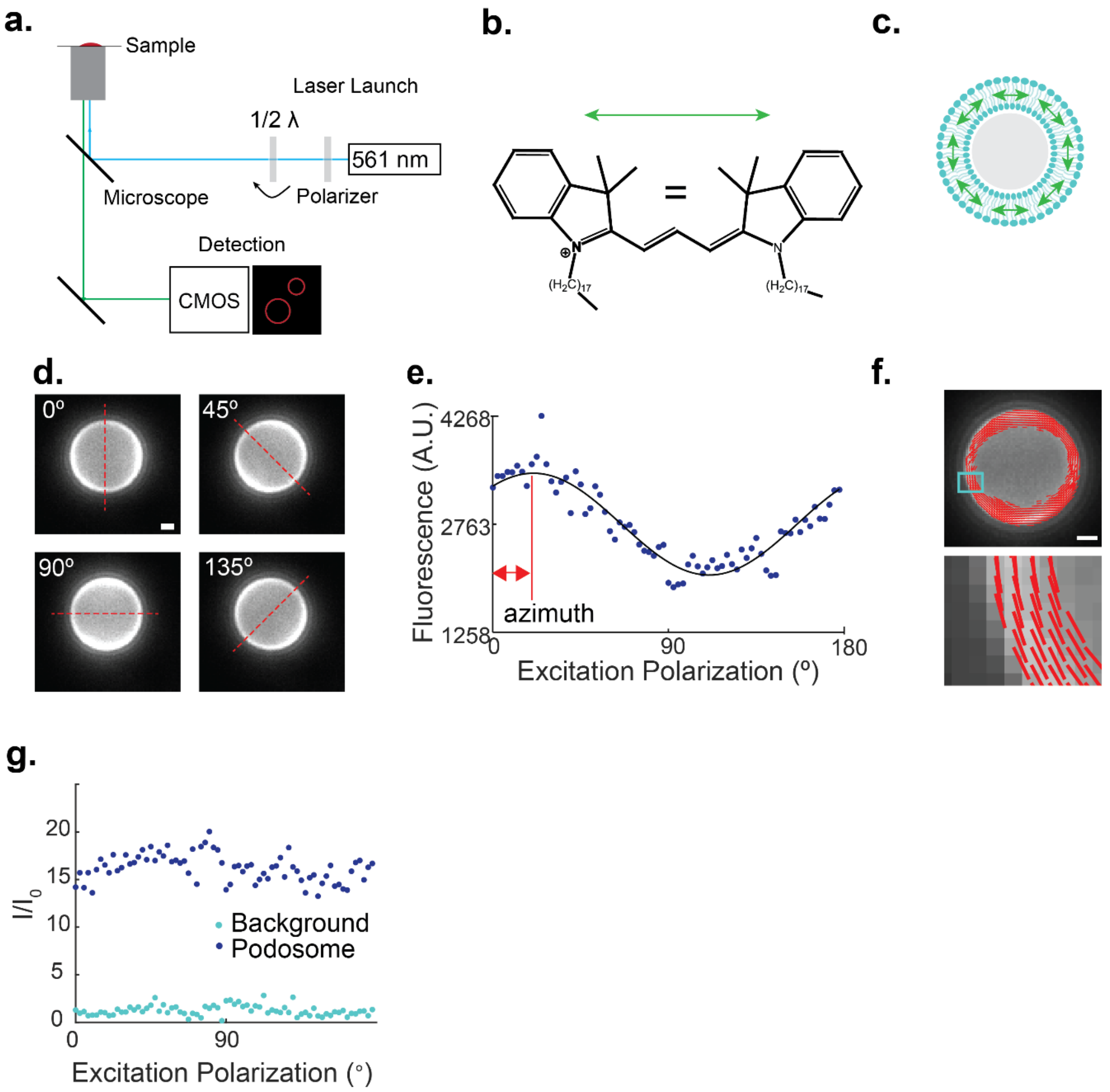

Supplementary Figure 16: MFM set-up and excitation-resolved polarization validation. (a) Optical configuration for MFM. The excitation polarization was varied using a ½ wave plate and a rotating polarizer. **(b,c)** To validate our MFM set-up, we imaged SLB-coated silica beads loaded with DiI. DiI is known to align parallel to the membrane[8]. The green arrow indicates the direction of the fluorophore's dipole. **(d)** Representative images of DiI loaded beads at varying excitation polarization angles (N=3). **(e)** Representative per-pixel plot of fluorescence intensity as a function of excitation polarization. The phase of the sinusoid (indicated by the red arrow) corresponds to the azimuthal angle, which is the measured orientation of the fluorophore's transition dipole moment. **(f)** Map of DiI orientation on an SLB-coated bead reveals that DiI is parallel to the SLB surface. **(g)** Representative per-pixel plot of normalized and bleach-corrected fluorescence intensity in podosome shown in Fig. 3b as a function of excitation polarization does not reveal significant variation in fluorescence intensity with varying excitation polarization orientation. Teal circles correspond to background fluorescence, and dark blue circles correspond to podosome fluorescence. Scale bars, 1 µm.

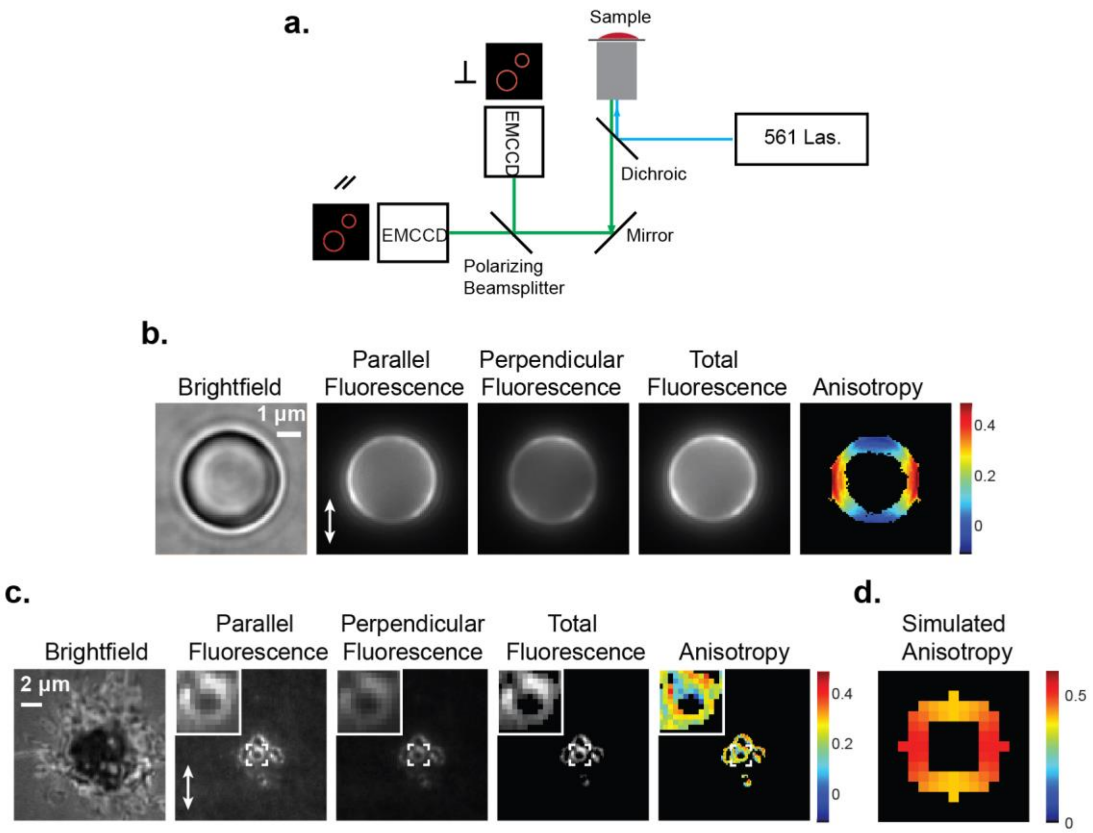

Supplementary Figure 17: Emission resolved fluorescence data does not indicate lateral organization of integrin forces in podosomes. (a) Optical configuration for emission resolved fluorescence polarization imaging. Tension probe fluorescence emission was split into parallel and perpendicular channels using a polarizing beamsplitter. **(b)** Representative DiI-loaded SLBs on 5 μm silica beads exhibit systematic variations in anisotropy around the bead perimeter (consistent with DiI alignment parallel to the SLB on the bead surface), validating emission resolved polarization imaging (N = 23 beads, 3 experiments). **(c)** Representative emission resolved polarization imaging of a podosome-forming cell (N=29 cells, 2 experiments). **(d)** Simulated podosome (radius = 1 μm) with a contractile ring of 20° (selected to approximate MFM data). In contrast with the anisotropy of DiI-doped SLBs on 5 μm beads, podosome integrin tension does not exhibit systematic spatial variations in anisotropy. White arrows indicate laser polarization.

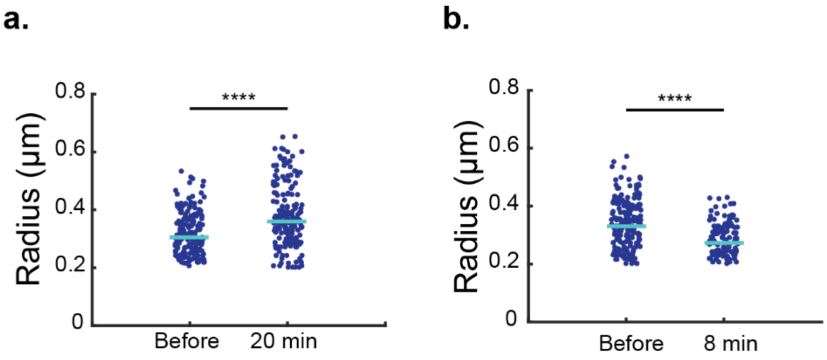

Supplementary Fig. 18: Actin polymerization and nonconventional myosin regulate podosome mechanics. (a, b) Podosome depletion radius before and after drug treatment with 50 µM Y27632 or 0.5 µM Jasplakinolide, respectively. Statistics were performed with a two-tailed Mann-Whitney test. Teal lines represent the median. Outliers were excluded (median ± 3 scaled median absolute deviations). Each condition contains data from at least 91 individual podosomes with an eccentricity of <0.7 from at least 30 cells, 3 experiments. Source data are provided as a Source Data file.

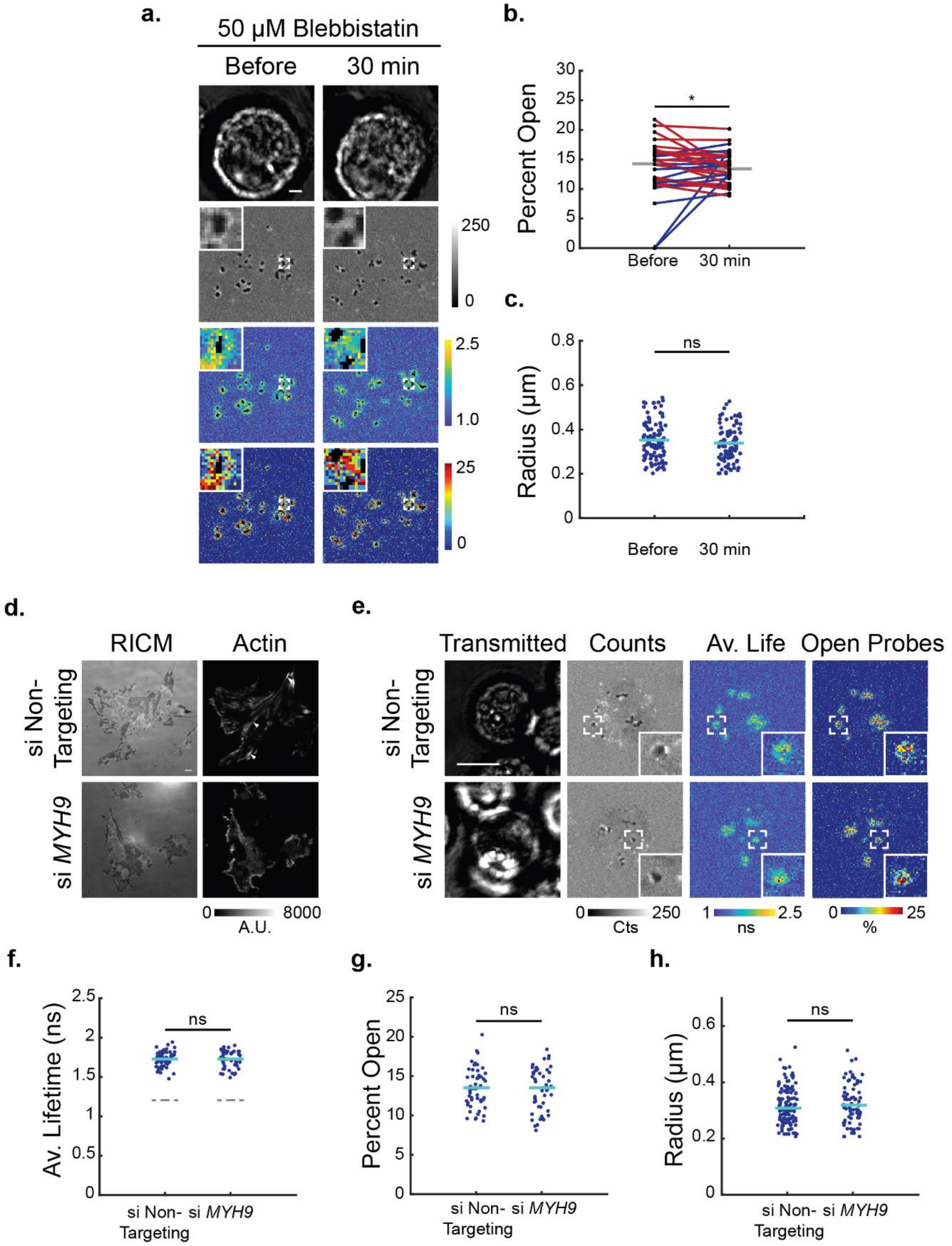

Supplementary Fig. 19: Myosin IIa is dispensable in podosome ring force generation. (a) Representative images of a podosome-forming cell before and after treatment with 50 μM blebbistatin. **(b)** Average percent open probes in podosomes per cell before and after

blebbistatin treatment. Blue and red lines represent an increase or decrease in percent open probes per cell, respectively. Grey horizontal lines represent the mean percent open probes. Statistics were performed with a two-tailed paired Students t-test. 31 cells, 4 experiments. Scale Bar, 2.5 μm. **(c)** Podosome depletion radius before and after drug treatment with 50 μM Blebbistatin. Statistics were performed with a two-tailed unpaired t-test. Teal bars represent the mean. Outliers were excluded (Median ± 3 scaled median absolute deviations). Each graph contains data from at least 69 individual podosomes with an eccentricity of <0.7, 4 experiments. **(d)** Representative images of actin staining of cells treated with nontargeting siRNA or si*MYH9* after ~12 hours on glass. *MYH9* knockdown cells had impaired focal adhesion formation. Representative focal adhesions are marked by white arrows. Scale Bar, 5 μm. **(e)** Representative MT-FLIM images of podosome-forming transfected cells on an SLB. Scale Bar, 5 μm. **(f)** Average fluorescence lifetime in podosome regions of control and si*MYH9* cells. Teal bars represent the mean, and grey bars represent the mean SLB fluorescence lifetime. At least 47 cells per condition were analyzed, 3 experiments. Statistics were performed with a two-tailed unpaired t test. **(g)** Average percent open in podosome regions of control and si*MYH9* cells. Teal bars represent the median. At least 47 cells per condition were analyzed, 3 experiments. Statistics were performed with a two-tailed Mann-Whitney Test. **(h)** Podosome depletion radius in siNT and si*MYH9* cells. Statistics were performed with a two-tailed, Mann-Whitney Test. Outliers were excluded (median ± 3 scaled median absolute deviations). Teal bars represent the median. Each condition contains data from at least 74 individual podosomes with an eccentricity of <0.7, 3 experiments. ns, P>0.05, *P<0.05. Source data are provided as a Source Data file.

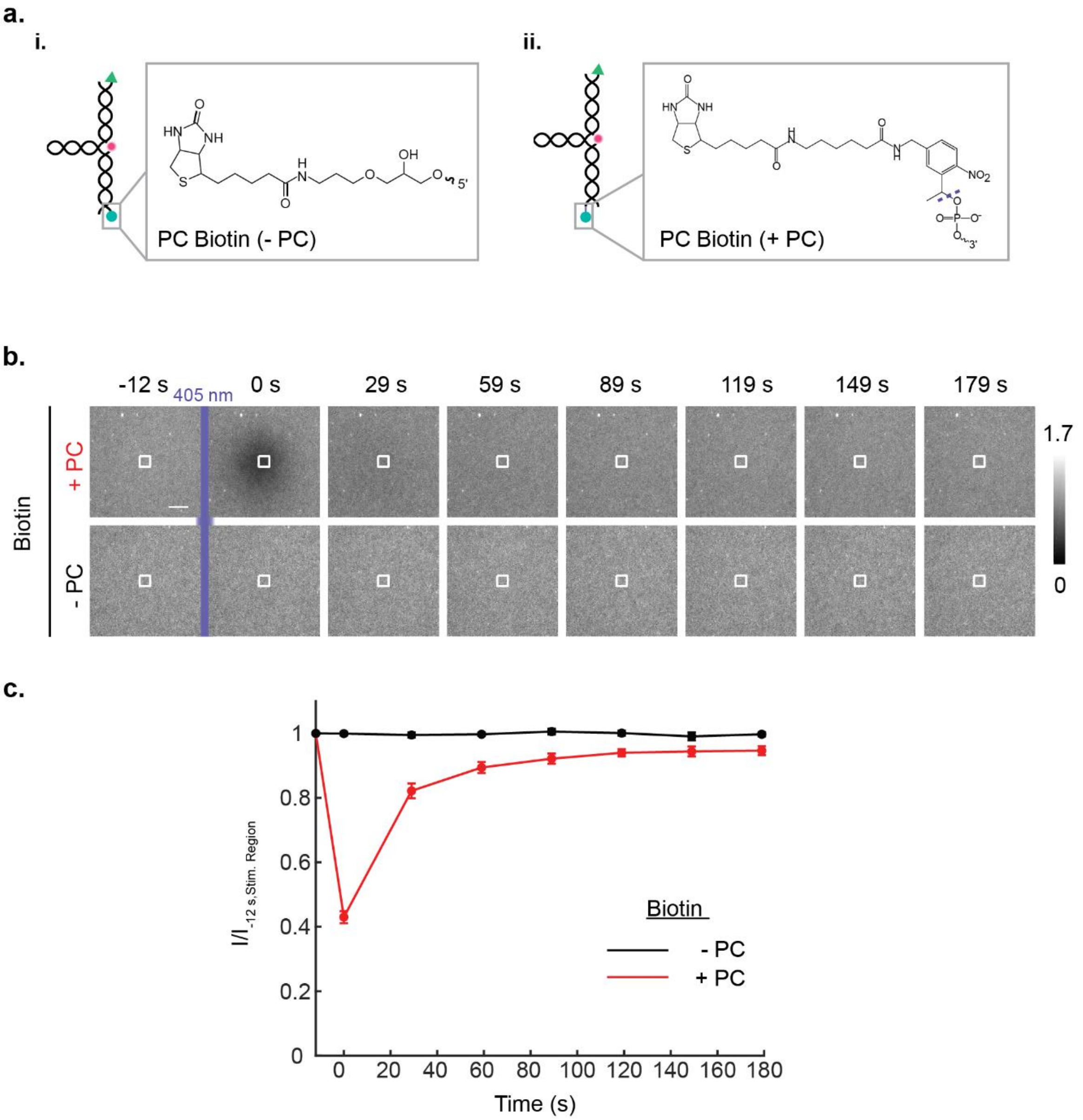

Supplementary Figure 20: Kinetics of fluorescence recovery after biotin photostimulation. (a) Representative images of SLBs with DNA probes attached to the SLB with either regular or photocleavable biotin. **(b)** SLBs were photostimulated in the red box as described in **Fig. 5c**. Scale bar, 5 μm. **(c)** Quantification of fluorescence in the photostimulation region indicated in **(a)**. DNA with a photocleavable biotin was ~60% released. SLBs recovered by lateral diffusion. Red lines represent SLBs with photocleavable biotin (+PC); black lines represent control SLBs with regular biotin (-PC). SLBs lacking a photocleavable biotin group were not released or photobleached under these illumination conditions. Data represent the mean ± s.e.m. (error bars), 3 experiments. Source data are provided as a Source Data file.

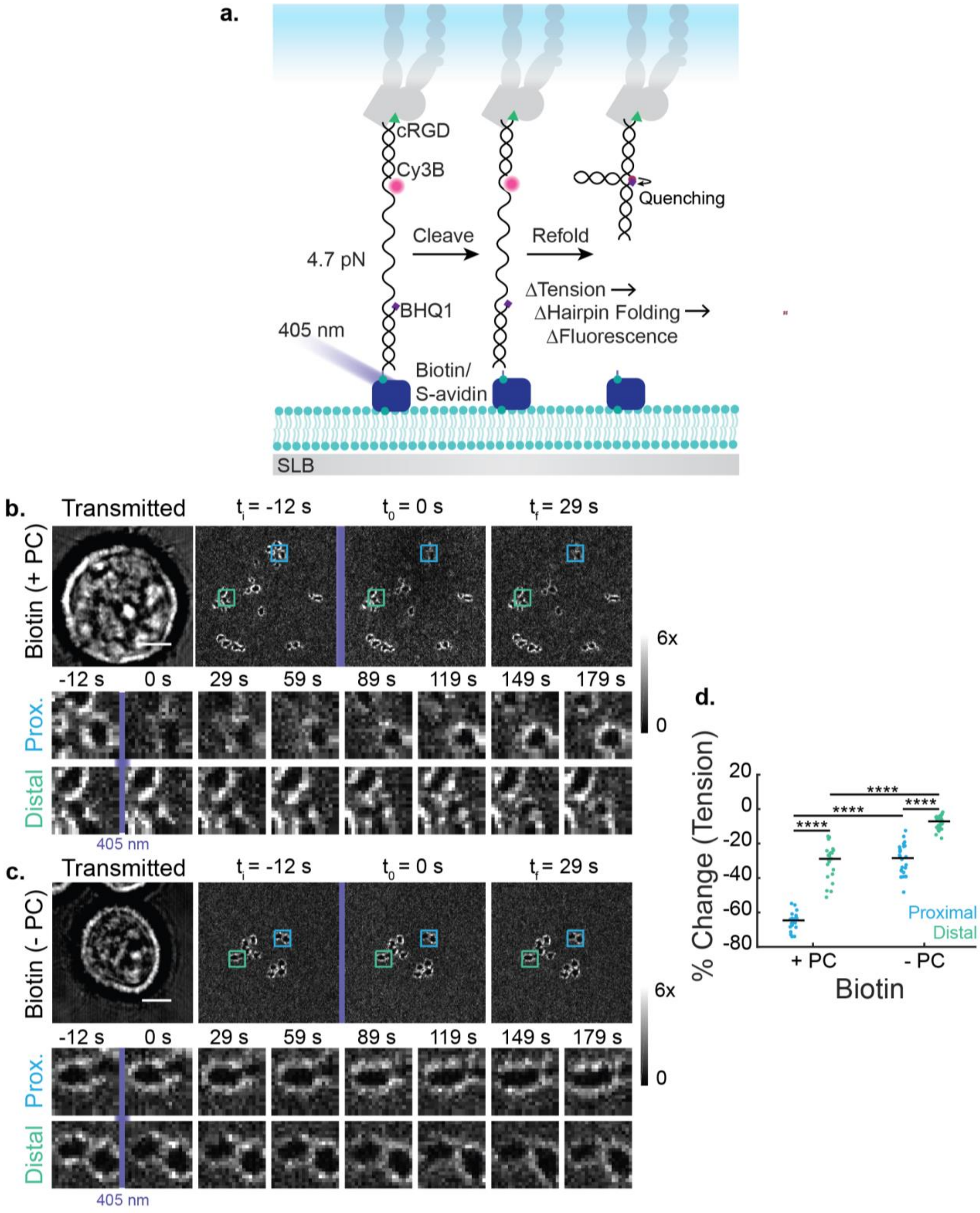

Supplementary Figure 21: Integrin tension is released following PCB photostimulation. **(a)** Schematic of photocleavable tension probes. When the biotin anchor is cleaved with a 405 nm laser, the probe detaches from the bilayer and refolds, severing the podosome's mechanical connection to the SLB and quenching Cy3B fluorescence. **(b,c)** Representative time-lapse acquisitions of podosome tension proximal and distal to the site of photostimulation on

substrates with a photocleavable biotin group and with a regular biotin anchor. **(d)** Percent change in tension proximal (blue) and distal (green) to photocleavage on probes containing regular biotin and PCB. Statistics were performed with a 2-way ANOVA. Each group contained at least 23 cells, 3 experiments. ****P<0.0001. All scale bars, 5 μm. Source data are provided as a Source Data file.

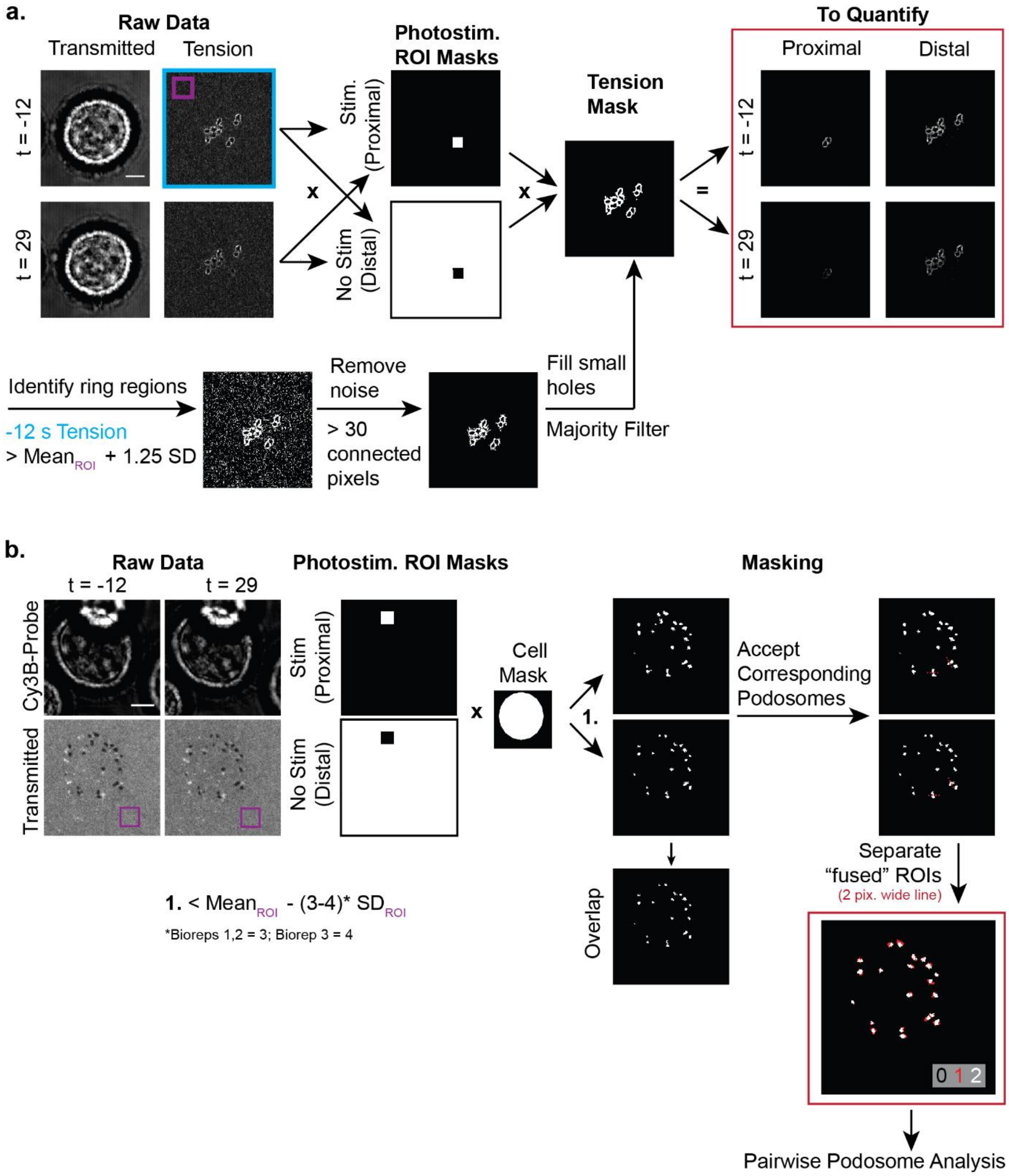

Supplementary Figure 22: Analysis protocol for photocleavable biotin experiments (a) Flowchart for PCB tension analysis. Masks were generated from the -12 s image, because the signal-to-noise ratio was not sufficiently high to reliably identify photo-stimulated podosomes at later time points. First, tension regions were identified by intensity thresholding of the -12 s pre-stimulation image. Then, small objects in the background were removed, and a majority filter was used to fill any single pixel holes in rings. The tension mask was multiplied by both the raw tension signal and by the photostimulaton masks exported from Nikon Elements to produce quantifiable tension maps for proximal and distal regions of the cell. Proximal regions corresponded to the 7 μm^2 region that was photocleaved. **(b)** Analysis flowchart for PCB protrusion experiments. To generate masks of podosome depletion, the fluorescence images at -12 s and 29 s were intensity thresholded and multiplied by masks of the cell area, determined by the transmitted light, and by the photostimulation mask, which was exported from Nikon Elements. The cell area mask served to remove any holes in SLB in the field of view. Podosome masks were overlapped, and any podosomes in the original mask that aligned with the overlap mask were accepted. Clear podosome doublets were split in two using a 2-pixel line. Single podosomes were processed. A small percentage of podosomes could not be clearly distinguished from their neighbors or were double-counted in the overlap criteria; these data were excluded.

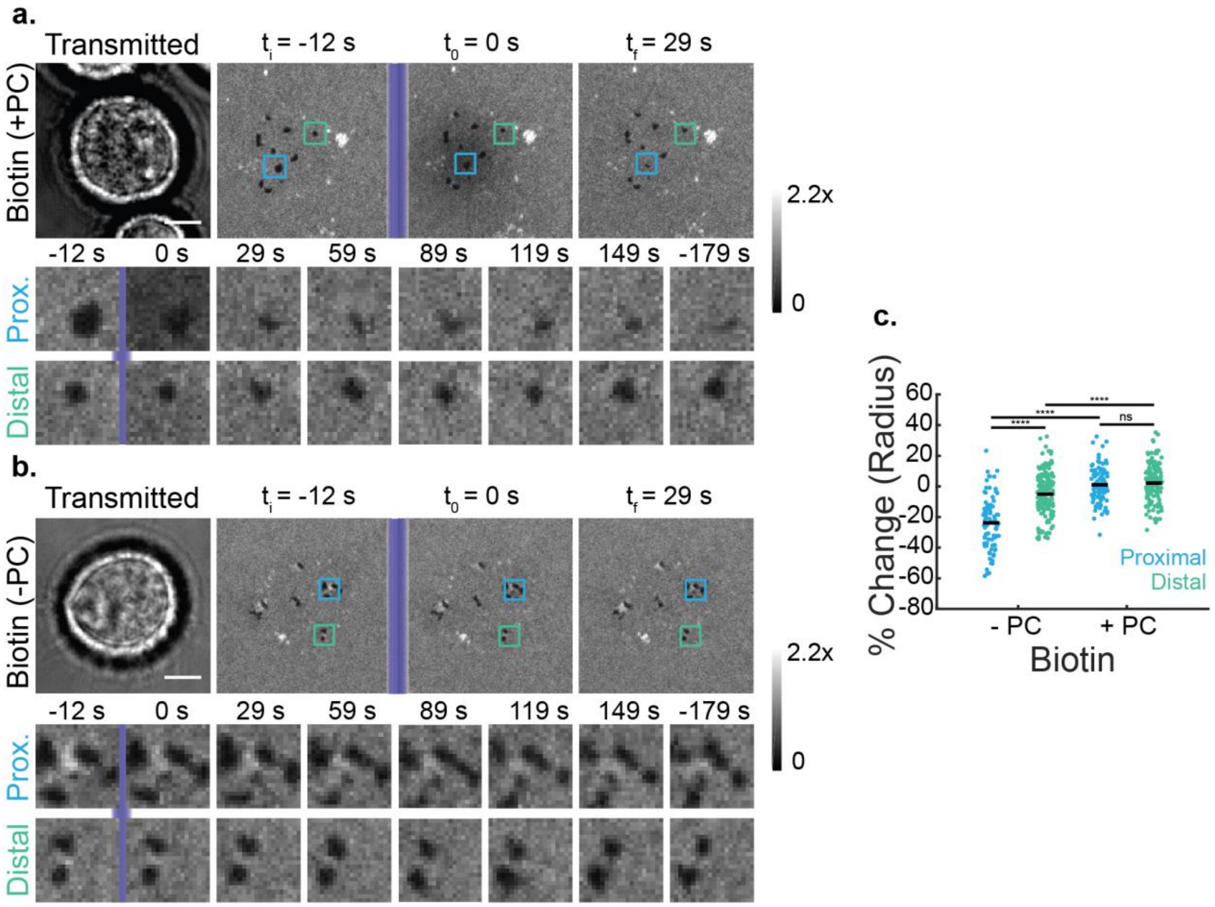

Supplementary Figure 23: Podosome protrusion is primarily perturbed at the site of photocleavage. (a,b) Representative time-lapse acquisitions of probe density proximal and distal to the site of photostimulation on substrates with a photocleavable biotin group and with a regular biotin anchor. **(c)** Percent change depletion radius proximal (blue) and distal (green) to photocleavage on probes containing regular biotin and PCB at t = 29 s. Statistics were performed with a 2-way ANOVA. Each group contained at least 79 podosomes across 3 experiments. Outliers were excluded (median ± 3 scaled median absolute deviations). ns P>0.05, ****P<0.0001. Scale Bar, 5 μm. Source data are provided as a Source Data file.

CELL CHECK

Species-specific PCR Evaluation

Species	1
mouse	+
rat	-
human	-
Chinese hamster	-
African green monkey	-

Marker Analysis

Marker Name	1	
	Sample Results	NIH/3T3 (CRL-1658)
MCA-4-2	19.3	19.3, 20.3
MCA-5-5	14, 15	14, 15
MCA-6-4	14.3	14.3
MCA-6-7	12	12
MCA-9-2	15, 16	15, 16
MCA-12-1	20	20
MCA-15-3	20.3	20.3
MCA-18-3	17, 18, 19	17, 19
MCA-X-1	25	25

Sample ID	Remarks
1	The sample was confirmed to be of mouse origin and no mammalian interspecies contamination was detected. A genetic profile was generated for the sample by using a panel of STR markers for genotyping.<br><br>The sample profile has minor genetic changes (loss of an allele at marker MCA-4-2 and an addition of an allele at marker MCA-18-3), but is otherwise identical to the genetic profile established for this cell line.

Supplementary Figure 24: NIH-3T3 cell profile. Cell profile by IDEXX Bioanalytics. Short tandem repeat analysis of NIH-3T3 cells reveals an 85% match to the standard, confirming cell line identity[9].

Supplementary References:

1. de Gennes P-G. Maximum pull out force on DNA hybrids. *Comptes Rendus de l'Académie des Sciences - Series IV - Physics* **2**, 1505-1508 (2001).

2. Hatch K, Danilowicz C, Coljee V, Prentiss M. Demonstration that the shear force required to separate short double-stranded DNA does not increase significantly with sequence length for sequences longer than 25 base pairs. *Physical review E, Statistical, nonlinear, and soft matter physics* **78**, 011920 (2008).

3. Zhang Y, Ge C, Zhu C, Salaita K. DNA-based digital tension probes reveal integrin forces during early cell adhesion. *Nature communications* **5**, 5167 (2014).

4. Holoubek A*, et al.* Monitoring of nucleophosmin oligomerization in live cells. *Methods and Applications in Fluorescence* **6**, 035016 (2018).

5. Anthony NR, Mehta AK, Lynn DG, Berland KM. Mapping amyloid-beta(16-22) nucleation pathways using fluorescence lifetime imaging microscopy. *Soft matter* **10**, 4162-4172 (2014).

6. Ostašov P, Sýkora J, Brejchová J, Olżyńska A, Hof M, Svoboda P. FLIM studies of 22- and 25-NBD-cholesterol in living HEK293 cells: Plasma membrane change induced by cholesterol depletion. *Chemistry and Physics of Lipids* **167-168**, 62-69 (2013).

7. van den Dries K*, et al.* Dual-color superresolution microscopy reveals nanoscale organization of mechanosensory podosomes. *Molecular biology of the cell* **24**, 2112-2123 (2013).

8. Oreopoulos J, Yip CM. Probing membrane order and topography in supported lipid bilayers by combined polarized total internal reflection fluorescence-atomic force microscopy. *Biophysical journal* **96**, 1970-1984 (2009).

9. Reid YS, D; Riss, T; and Minor, L. Assay Guidance Manual: Authentication of Human Cell Lines by STR DNA Profiling. Eli Lily & Company and National Center for Advancing Translational Sciences (2013).
