# Supplementary material for: DNA mechanotechnology reveals that integrin receptors apply pN forces in podosomes on fluid substrates
Source: Nat Commun. 2019 Oct 18;10:4507. doi: 10.1038/s41467-019-12304-4 (PMC6800454; doi:10.1038/s41467-019-12304-4)
Supplement: Supplementary file 5 — Reporting Summary [file 41467_2019_12304_MOESM5_ESM.pdf]

nature research

Corresponding author(s): Khalid Salaita

Last updated by author(s): Jul 4, 2019

Reporting Summary

Nature Research wishes to improve the reproducibility of the work that we publish. This form provides structure for consistency and transparency in reporting. For further information on Nature Research policies, see Authors & Referees and the Editorial Policy Checklist.

Statistics

For all statistical analyses, confirm that the following items are present in the figure legend, table legend, main text, or Methods section.

n/a	Confirmed	
☐	☒	The exact sample size (*n*) for each experimental group/condition, given as a discrete number and unit of measurement
☐	☒	A statement on whether measurements were taken from distinct samples or whether the same sample was measured repeatedly
☐	☒	The statistical test(s) used AND whether they are one- or two-sided<br>*Only common tests should be described solely by name; describe more complex techniques in the Methods section.*
☒	☐	A description of all covariates tested
☐	☒	A description of any assumptions or corrections, such as tests of normality and adjustment for multiple comparisons
☐	☒	A full description of the statistical parameters including central tendency (e.g. means) or other basic estimates (e.g. regression coefficient) AND variation (e.g. standard deviation) or associated estimates of uncertainty (e.g. confidence intervals)
☐	☒	For null hypothesis testing, the test statistic (e.g. *F*, *t*, *r*) with confidence intervals, effect sizes, degrees of freedom and *P* value noted<br>*Give P values as exact values whenever suitable.*
☒	☐	For Bayesian analysis, information on the choice of priors and Markov chain Monte Carlo settings
☒	☐	For hierarchical and complex designs, identification of the appropriate level for tests and full reporting of outcomes
☐	☒	Estimates of effect sizes (e.g. Cohen's *d*, Pearson's *r*), indicating how they were calculated

Our web collection on statistics for biologists contains articles on many of the points above.

Software and code

Policy information about availability of computer code

Data collection	FLIM imaging was performed with SymPhoTime 64 2.1.3813. Epifluorescence, TIRFM, and emission-resolved polarization imaging were performed in Nikon Elements 4.40.00 or Nikon Elements 4.13.05. Excitation-resolved polarization imaging was performed in Nikon Elements v5.1.
Data analysis	MALDI-TOF data was analyzed with flexAnalysis 3.4. Image analysis was performed in MATLAB 2018a, ImageJ, FIJI, and GraphPad as described in the methods section and supplementary figures. Absorbance spectroscopy was processed in MATLAB and GraphPad Prism 7. FLIM histograms were generated in SymPhoTime 6 2.1.3813. Statistics were performed in MATLAB and GraphPad.

For manuscripts utilizing custom algorithms or software that are central to the research but not yet described in published literature, software must be made available to editors/reviewers. We strongly encourage code deposition in a community repository (e.g. GitHub). See the Nature Research guidelines for submitting code & software for further information.

Data

Policy information about availability of data

All manuscripts must include a data availability statement. This statement should provide the following information, where applicable:

- Accession codes, unique identifiers, or web links for publicly available datasets
- A list of figures that have associated raw data
- A description of any restrictions on data availability

The data that support the findings of this study are available from the corresponding author upon reasonable request.

Field-specific reporting

Please select the one below that is the best fit for your research. If you are not sure, read the appropriate sections before making your selection.

☒ Life sciences ☐ Behavioural & social sciences ☐ Ecological, evolutionary & environmental sciences

For a reference copy of the document with all sections, see nature.com/documents/nr-reporting-summary-flat.pdf

Life sciences study design

All studies must disclose on these points even when the disclosure is negative.

Sample size	Sample size was not predetermined. All cell experiments were performed in triplicate, with the exception of the data shown in SI 9 and 15, which was performed in duplicate. Surface characterization was performed at least twice, with most characterizations performed at least three times. Data were reported on a per cell or per podosome basis depending on the relevant biological question.
Data exclusions	Only experiments with high surface density to support cell spreading and podosome formation and high photon counts and imaged with a count rate of ~4-6% for a high signal-to-noise ratio were quantitatively analyzed. An exception to this was SI8, which was a standalone cell-free data set and had lower DNA density. Cells on regions of the bilayer that were photobleached or that contained significant defects that prohibited clear identification and analysis of podosomes were not processed. Cells that significantly extended beyond the field of view or were in poor focus were excluded. For MFM, MT-FLIM, and TGT experiments, a subset of a data was analyzed due to time constraints. This subset was based on image acquisition number (ie - For TGT experiments, images 1 - 20 were processed per bioreplicate). Outlier data points as determined by statistical testing (described in figure captions) were excluded. Podosomes were identified and selected as described in the methods section of the paper. Podosomes that did not meet the set threshold were not included.
Replication	A bioreplicate was defined as one flask or well of cells. A surface replicate was defined as a single SLB. Cell experiments were repeated at least three times, except for the data in figure S9 and S15, which were repeated twice. Surface characterization was repeated at least twice, with the majority of experiments repeated at least three times. Absorbance spectroscopy was repeated three times.
Randomization	To determine the percentage of cells forming podosomes in TGT experiments, regions of the SLB were randomly selected for imaging, with slight frame adjustments to maximize the number of cells in view. For all other experiments, the imaging frame was selected to contain podosome-forming cells.
Blinding	To confirm MYH9 knockdown with a stress fiber assay, images were correctly and blindly matched to treatment groups by three non authors. Image analysis was performed using semi-automated codes that treated control and experimental groups identically, as described in supplementary figures and methods.

Reporting for specific materials, systems and methods

We require information from authors about some types of materials, experimental systems and methods used in many studies. Here, indicate whether each material, system or method listed is relevant to your study. If you are not sure if a list item applies to your research, read the appropriate section before selecting a response.

Materials & experimental systems

n/a	Involved in the study	
☐	☒	Antibodies
☐	☒	Eukaryotic cell lines
☒	☐	Palaeontology
☒	☐	Animals and other organisms
☒	☐	Human research participants
☒	☐	Clinical data

Methods

n/a	Involved in the study	
☒	☐	ChIP-seq
☒	☐	Flow cytometry
☒	☐	MRI-based neuroimaging

Antibodies

Antibodies used	Vinculin Antibody SF9 647 (sc-73614 AF647, Santa Cruz Biotechnology), Phospho-Paxcillin (Tyr1888) Polyclonal Antibody (PA5-17828, Thermo-Fisher) followed by Alexa Fluor 555 goat anti-rabbit (A21147, Thermo Fisher), or Anti-Integrin β1 Antibody, clone MB1.2 (MAB1997, Sigma-Aldrich), followed by Alexa Fluor 647 goat anti-mouse IgG2b (γ2b) (Thermo-Fisher).
Validation	Vinculin antibody 7F9 was previously cited in 61 publications, which are listed on the manufacturer's website. Anti-Integrin β1 Antibody, clone MB1.2 publications are listed at the manufacturers website. PA5-17828 was previously used for IF in one publication (Kantarchi, et. al. PNAS, 2016). Secondary antibodies are previously cited in several publications, available on the manufacturers website.

Eukaryotic cell lines

Policy information about cell lines

Cell line source(s)	NIH 3T3, Myf (Destaing Lab)
Authentication	NIH 3T3 cells were authenticated by IDEXX Bioresearch. 3T3 cells exceeded the literature standard for authentication using STR profiling (Reid, et. al. Assay Guidance Manual, 2013), but showed slight genetic drift. Myf cells were not authenticated.
Mycoplasma contamination	Cells were not tested for mycoplasma contamination.
Commonly misidentified lines<br>(See ICLAC register)	This study did not utilize commonly misidentified cell lines.
