# Supplementary material for: DNA mechanotechnology reveals that integrin receptors apply pN forces in podosomes on fluid substrates
Source: Nat Commun. 2019 Oct 18;10:4507. doi: 10.1038/s41467-019-12304-4 (PMC6800454; doi:10.1038/s41467-019-12304-4)
Supplement: Supplementary file 6 — Description of Additional Supplementary Files [file 41467_2019_12304_MOESM6_ESM.pdf]

Title: Supplementary Movie 1
Description: Mechanical evolution of podosomes on an SLB. MT-FLIM video of the cell in Supplementary Fig. 14. FLIM images were acquired for 3 minutes and subdivided into three-minute intervals. Transmitted detector images were acquired every 3 minutes. Framerate, 10 frames-per-second. Scale bar, 5 µm.

Title: Supplementary Movie 2
Description: Podosomes on 12 pN TGTs are small and short-lived. Epifluorescence video of podosomes on an SLB presenting 12 pN TGTs. Images were acquired every 30 s. Framerate, 15 frames-per-second. Scale bar, 5 µm.

Title: Supplementary Movie 3
Description: Podosomes on 56 pN TGTs are large and long-lived. Epifluorescence video of podosomes on an SLB presenting 56 pN TGTs. Images were acquired every 30 s. Framerate, 15 frames-per-second. Scale bar, 5 µm.
